# Hearing Intervention, Social Isolation, and Loneliness: A Secondary Analysis of the ACHIEVE Randomized Clinical Trial

**DOI:** 10.1001/jamainternmed.2025.1140

**Published:** 2025-05-12

**Authors:** Nicholas S. Reed, Jinyu Chen, Alison R. Huang, James R. Pike, Michelle Arnold, Sheila Burgard, Ziheng Chen, Theresa Chisolm, David Couper, Thomas K. M. Cudjoe, Jennifer A. Deal, Adele M. Goman, Nancy W. Glynn, Theresa Gmelin, Lisa Gravens-Mueller, Kathleen M. Hayden, Christine M. Mitchell, Thomas Mosley, Esther S. Oh, James S. Pankow, Victoria A. Sanchez, Jennifer A. Schrack, Josef Coresh, Frank R. Lin

**Affiliations:** 1 Optimal Aging Institute, New York University Grossman School of Medicine, New York; 2 Department of Epidemiology, Johns Hopkins Bloomberg School of Public Health, Baltimore, Maryland; 3 Cochlear Center for Hearing and Public Health, Johns Hopkins Bloomberg School of Public Health, Baltimore, Maryland; 4 University of South Florida, Tampa; 5 Department of Biostatistics, Gillings School of Global Public Health, University of North Carolina, Chapel Hill; 6 Division of Geriatric Medicine and Gerontology, Department of Medicine, Johns Hopkins University School of Medicine, Baltimore, Maryland; 7 School of Health and Social Care, Edinburgh Napier University, Edinburgh, Scotland; 8 Department of Epidemiology, University of Pittsburgh School of Public Health, Pittsburgh, Pennsylvania; 9 Department of Social Sciences and Health Policy, Wake Forest University School of Medicine, Winston-Salem, North Carolina; 10 Department of Neurology, University of Mississippi Medical Center, Jackson; 11 Department of Medicine, University of Mississippi Medical Center, Jackson; 12 Division of Epidemiology and Community Health, University of Minnesota School of Public Health, Minneapolis

## Abstract

**Importance:**

Promoting social connection among older adults is a public health priority. Addressing hearing loss may reduce social isolation and loneliness among older adults.

**Objective:**

To describe the effect of a best-practice hearing intervention vs health education control on social isolation and loneliness over a 3-year period in the Aging and Cognitive Health Evaluation in Elders (ACHIEVE) study.

**Design, Setting, and Participants:**

This secondary analysis of a multicenter randomized controlled trial with 3-year follow-up was completed in 2022 and conducted at 4 field sites in the US (Forsyth County, North Carolina; Jackson, Mississippi; Minneapolis, Minnesota; Washington County, Maryland). Data were analyzed in 2024. Participants included 977 adults (aged 70-84 years who had untreated hearing loss without substantial cognitive impairment) recruited from the Atherosclerosis Risk in Communities study (238 [24.4%]) and newly recruited (de novo; 739 [75.6%]). Participants were randomized (1:1) to hearing intervention or health education control and followed up every 6 months.

**Interventions:**

Hearing intervention (4 sessions with certified study audiologist, hearing aids, counseling, and education) and health education control (4 sessions with a certified health educator on chronic disease, disability prevention).

**Main Outcomes and Measures:**

Social isolation (Cohen Social Network Index score) and loneliness (UCLA Loneliness Scale score) were exploratory outcomes measured at baseline and at 6 months and 1, 2, and 3 years postintervention. The intervention effect was estimated using a 2-level linear mixed-effects model under the intention-to-treat principle.

**Results:**

Among the 977 participants, the mean (SD) age was 76.3 (4.0) years; 523 (53.5%) were female, 112 (11.5%) were Black, 858 (87.8%) were White, and 521 (53.4%) had a Bachelor’s degree or higher. The mean (SD) better-ear pure-tone average was 39.4 dB (6.9). Over 3 years, mean (SD) social network size reduced from 22.6 (11.1) to 21.3 (11.0) and 22.3 (10.2) to 19.8 (10.2) people over 2 weeks in the hearing intervention and health education control arms, respectively. In fully adjusted models, hearing intervention (vs health education control) reduced social isolation (social network size [difference, 1.05; 95% CI, 0.01-2.09], diversity [difference, 0.19; 95% CI, 0.02-0.36], embeddedness [difference, 0.27; 95% CI, 0.09-0.44], and reduced loneliness [difference, −0.94; 95% CI, −1.78 to −0.11]) over 3 years. Results were substantively unchanged in sensitivity analyses that incorporated models that were stratified by recruitment source, analyzed per protocol and complier average causal effect, or that varied covariate adjustment.

**Conclusions and Relevance:**

This secondary analysis of a randomized clinical trial indicated that older adults with hearing loss retained 1 additional person in their social network relative to a health education control over 3 years. While statistically significant, it is unknown whether observed changes in social network are clinically meaningful, and loneliness measure changes do not represent clinically meaningful changes. Hearing intervention is a low-risk strategy that may help promote social connection among older adults.

**Trial Registration:**

ClinicalTrials.gov Identifier: NCT03243422

## Key Points

**Question:**

What is the effect of hearing intervention on social isolation and loneliness over 3 years in older adults with previously untreated hearing loss?

**Findings:**

In this secondary analysis of the Aging and Cognitive Health Evaluation in Elders study, a randomized clinical trial of 977 older adults with untreated hearing loss, hearing intervention participants retained a mean of 1 additional person in their social network and experienced positive effects in social network diversity and quality and loneliness measures relative to health education control over 3 years.

**Meaning:**

The study results suggest that hearing intervention is a scalable, low-risk strategy that if implemented broadly may allow for a large population-level reduction in social isolation and loneliness.

## Teaser

This secondary analysis of randomized clinical trial examines the effect of a best-practice hearing intervention vs health education control on social isolation and loneliness.

## Introduction

Recent reports from the National Academies^1^ and Surgeon General of the US^2^ highlight the public health importance of and need for solutions to promote social connection and combat social isolation (an objective construct of fewer or less frequent social contact) and loneliness (a subjective construct of perceived isolation) in older adults. In the US, a quarter of older adults are socially isolated, while a third report feeling lonely.^1,3^ Social isolation and loneliness are associated with adverse aging outcomes, including morbidity, poor health resource utilization, dementia, and mortality.^4,5,6,7,8,9,10^ Recent health economic estimates suggest that social isolation among older adults accounts for $6.7 billion in excess annual Medicare spending.^11^

Addressing hearing loss, which is prevalent in two-thirds of adults older than 70 years,^12^ may represent an approach to reducing social isolation and loneliness among older adults. Hearing plays a vital role in communication and social connections. Large systematic reviews reported consistent associations between hearing loss and social isolation and loneliness across observational studies, but the role of hearing intervention in helping mitigate outcomes remains unclear.^13,14,15^ The Aging and Cognitive Health Evaluation in Elders (ACHIEVE; NCT03243422)^16,17^ study was a large, prospective clinical trial that tested the effects of hearing intervention vs health education control on 3-year cognitive decline (primary outcome). Measures of social isolation and loneliness were included as exploratory outcomes in this trial. We report results from a prespecified secondary analysis of the ACHIEVE study that investigated the effect of a hearing intervention vs health education control on social isolation and loneliness among older adults over a 3-year period.

## Methods

### Study Design and Participants

**Figure 1.**
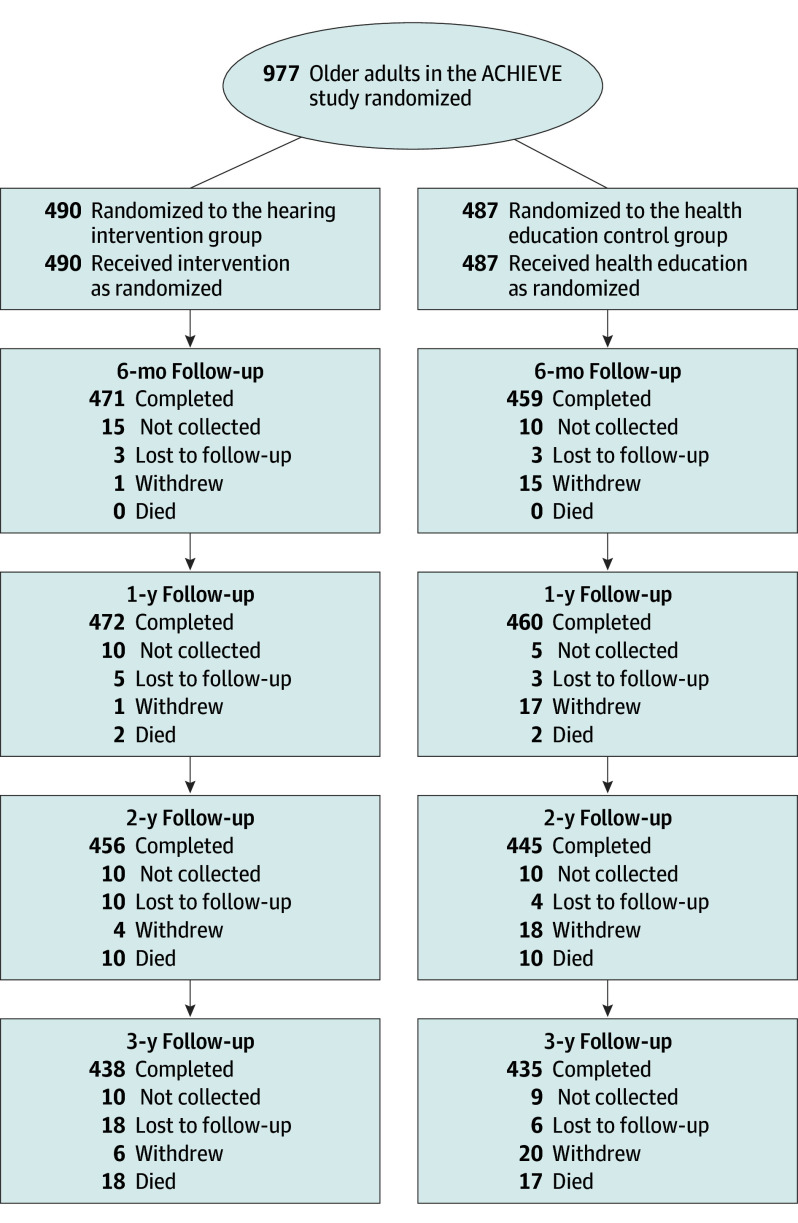
Trial Profile The status of participants was defined as completed if they completed either the Cohen Social Network Index or the UCLA Loneliness Score examination. ACHIEVE indicates Aging and Cognitive Health Evaluation in Elders.

The ACHIEVE study is a 3-year multicenter, parallel group, unblinded randomized clinical trial among community-dwelling older adults (Supplement 1 and Supplement 2; Figure 1). The ACHIEVE study was partially embedded within the scientific and physical infrastructure of the Atherosclerosis Risk in Communities (ARIC) study, a prospective longitudinal study of adults aged 45 to 64 years (initially recruited from 1987-1989 [N = 15 792]) from 4 US communities (Forsyth County, North Carolina; Jackson, Mississippi; Minneapolis, Minnesota; and Washington County, Maryland) and who have been followed up to the present day. This study followed the Consolidated Standards of Reporting Trials (CONSORT) reporting guideline.

At each study site, participants were recruited from the ARIC cohort and newly recruited (de novo) from the surrounding communities.^17^ The primary eligibility criteria for the study were age (70-84 years); living situation (community dwelling; not planning to move from the area); English language fluency; adult-onset bilateral hearing loss as defined by better-ear 4-frequency pure-tone average [PTA] at 500, 1000, 2000, and 4000 Hz between 30 to 70 dB, which is within the World Health Organization mild to moderate hearing loss range (better-ear PTA represents an approximation of the lowest decibel level at which a human can detect frequencies most important for speech); not having substantial cognitive impairment (Mini-Mental State Examination score of ≥23 for participants with a high school degree or less and ≥25 for those with some college education or more); no difficulty with 2 or more activities of daily living (ie, getting in/out of bed or chairs, bathing, dressing, eating, and toileting); presenting a visual acuity better than 20/63 on the MNREAD (University of Minnesota) acuity chart; no hearing aid use within the prior year; no concurrent enrollment in studies focused on auditory or cognitive exposures or outcomes; and a willingness to be randomized and participate in study interventions.

The ACHIEVE study was approved by the institutional review boards of all participating study sites and academic centers. Participants provided written informed consent. An independent data and safety monitoring board met every 6 months to review study progress. Articles offering comprehensive details on the design, methods, recruitment procedures, and baseline population characteristics have been previously published.^16,17,18,19^

### Procedures

Participants were randomly assigned 1:1 using permuted block randomization as stratified by hearing loss severity (PTA <40 dB or ≥40 dB), recruitment source (ARIC or de novo), and field site to a hearing intervention or a successful aging health education control. Eligible spousal pairs or partners were randomly assigned as a unit as stratified by recruitment source and field site. Intervention assignment was unmasked due to the evident nature of wearing hearing aids. However, steps to avoid bias were taken, including masking participants to the study hypothesis, stressing equipoise in the intervention and control, notifying participants that they would be offered the other intervention at the end of the 3-year period, and masking accumulating trial data from study investigators and staff.

The hearing intervention was developed based on evidence-based best practices developed during the feasibility and pilot phases of the ACHIEVE study.^20,21,22^ Briefly, the hearing intervention was characterized by four 1-hour sessions with an audiologist every 1 to 3 weeks following randomization. Sessions included fitting and verifying hearing aids to prescriptive targets using real ear measurements, provision of hearing assistive technologies to enhance hearing aid use in specific situations, extensive orientation and instruction on device use, communication strategies training, and counseling on self-efficacy and expectation management. Booster visits occurred every 6 months.

The health education control consisted of individual sessions with a certified health educator to administer the evidence-based 10 Keys to Healthy Aging^23^ program that focused on topics relevant to chronic condition management and disability prevention among older adults and has been used as an attention control intervention in previous trials.^24,25^ Sessions were tailored to the participant and included goal setting, educational counseling, activities, and 5- to 10-minute upper body extremity stretching. The social contact key was replaced with a caregiving and health key to avoid crossover effects with the proposed mechanism of hearing intervention on the primary outcome (cognitive decline). To parallel the staff contact of the hearing intervention, participants completed four 1-hour sessions every 1 to 3 weeks following randomization and returned for 6-month booster visits.

After baseline assessment, randomization, and completion of the intervention, participants were assessed in person during semiannual visits. During the COVID-19 pandemic period, visits continued with phone-based intervention booster sessions^16^ and phone-based assessments of study outcomes.

### Outcomes

Social isolation was measured by the Cohen Social Network Index,^6^ which measures 3 aspects of social network: size, diversity, and embeddedness. Participants were asked about regular contact (at least once every 2 weeks) with individuals across 12 social roles (eg, spouse, child, close friend, and neighbors) and 8 social network domains (eg, family, friends, and work). Social network size (score range, 0-84) was measured by the total number of people within each social role with whom the participant had regular contact. To avoid overinflation of social network size, the number of individuals identified within each social role was capped at 7 individuals. Social network diversity (score range, 0-12) describes the various social roles and relationships an individual participates in and was measured by the number of different regular contact social roles (eg, parent, child, and neighbor) an individual has. Social network embeddedness (score range, 0-8) describes the depth of an individual’s engagement in different social network domains and was measured by the number of social network domains (eg, family, friends, and work) in which the participant remains active (regular contact with 4 or more individuals). Loneliness was measured using the 20-item UCLA Loneliness Scale^26^ and analyzed as a continuous score. Social isolation and loneliness were measured at baseline and at 6 months and 1-, 2-, and 3-year follow-up visits.

### Covariates

Time-invariant covariates measured at baseline included age, sex (male and female), education (less than high school, completed high school, or some college or more), hearing loss severity (4-frequency PTA for the better-hearing ear), speech-in-noise perception (QuickSIN), hearing-related quality of life (Hearing Handicap Inventory for the Elderly, screening version), marital status (self-reported married vs not married), living alone (yes or no), global cognition, depressive symptoms (Center for Epidemiologic Studies Depression Scale), antidepressant use (yes or no), field site, and whether the participant was part of a recruited spousal pair. Time-varying covariates were created to address the potential effect of the COVID-19 global pandemic and related lockdowns. The first time-varying covariate was binary and introduced an immediate effect when a public health emergency was declared in the US on March 13, 2020. The second time-varying covariate was continuous and modeled the gradual easing of COVID-19–related restrictions by specifying a spline on June 30, 2021, when in-person data collection resumed.

### Statistical Analysis

The study population was characterized by descriptive statistics as stratified by randomization and recruitment source. The effect of the hearing intervention on 3-year changes in social isolation (as measured by social network size, diversity, and embeddedness) and loneliness was estimated under the intention-to-treat principle using a 2-level linear mixed-effects model with an unstructured covariance matrix, random intercept, and random slope. Restricted maximum likelihood with a Kenward-Roger correction was used to generate parameter estimates and 95% CIs. Models included randomization, time from baseline, the interaction between randomization and time, time-invariant and time-varying covariates, and the interaction between time and each time-invariant covariate. Missing measurements were addressed using multiple imputation by chained equations.

A series of sensitivity analyses were performed to assess the robustness of the results. The first sensitivity analysis examined whether the intervention effect differed by recruitment source (ARIC or de novo) by including a 3-way interaction between randomization, recruitment source, and time to primary models and by conducting analyses stratified by recruitment source. The second sensitivity analysis replicated the primary analysis but removed time-varying covariates designed to account for the effect of the COVID-19 pandemic. The third sensitivity analysis generated per protocol and complier average causal effect estimates. Lastly, we analyzed the intervention effect stratified by self-reported sex (male and female) due to sex differences in social and loneliness measures.^1^ All analyses were conducted in SAS, version 9.4 (SAS Institute) except for multiple imputation (Stata, version 18.0; StataCorp).

## Results

### Participants

At baseline, the mean (SD) age among participants (N = 977) was 76.3 (4.0) years, and the mean (SD) better-ear pure-tone average was 39.4 (6.9) dB HL (Table). Of the cohort participants, 523 (53.5%) were female, 858 (87.8%) were self-reported White, 602 (61.6%) were married, and 293 (30.0%) lived alone. Baseline participant characteristics were similar between the hearing intervention (490 [50.2%]) and health education control (487 [49.8%]). However, participants recruited from the ARIC cohort (238 [24.4%]) were slightly older and more likely to be female, Black, and live alone (eTable 1 in Supplement 3) compared with the de novo cohort (739 [75.6%]).

**Table.** Demographic and Clinical Characteristics at Baseline of 977 Aging and Cognitive Health Evaluation in Elders Study Participants Stratified by Randomly Assigned Treatment

Characteristic	No./total No. (%)	
	Control (n = 487)	Intervention (n = 490)
Age, mean (SD), y	76.5 (4.0)	76.1 (3.9)
Sex		
Female	259/487 (53.2)	264/490 (53.9)
Male	228/487 (46.8)	226/490 (46.1)
Race		
Black	59/487 (12.1)	53/490 (10.8)
White	424/487 (87.1)	434/490 (88.6)
Other	4/487 (0.8)	3/490 (0.6)
Center		
Forsyth County, North Carolina	119/487 (24.4)	117/490 (23.9)
Jackson, Mississippi	123/487 (25.3)	120/490 (24.5)
Minneapolis, Minnesota	116/487 (23.8)	120/490 (24.5)
Washington County, Maryland	129/487 (26.5)	133/490 (27.1)
Education		
<High school	18/487 (3.7)	19/489 (3.9)
High school, GED, or vocational school	212/487 (43.5)	206/489 (42.1)
Some college, graduate, or professional school	257/487 (52.8)	264/489 (54.0)
Better-ear pure-tone average, mean (SD), db HL	39.3 (6.7)	39.5 (7.1)
Quick speech in noise average score, mean (SD)	18.4 (5.0)	18.5 (5.4)
Hearing Handicap Inventory for Elderly score		
None (0-8)	154/485 (31.8)	150/485 (30.9)
Mild-moderate (10-24)	247/485 (50.9)	240/485 (49.5)
Severe (26-40)	84/485 (17.3)	95/485 (19.6)
Marital status	308/487 (63.2)	294/490 (60.0)
Participant part of a recruited spousal pair	44/487 (9.0)	46/490 (9.4)
Lives alone	137/484 (28.3)	153/484 (31.6)
CES-Depression Scale score, mean (SD)	2.5 (2.4)	2.5 (2.6)
Use of an antidepressant	66/487 (13.6)	66/490 (13.5)
Global cognition, mean (SD)^a^	−0.011 (0.902)	0.012 (0.949)

Abbreviations: CES, Center for Epidemiologic Studies; GED, general educational development credential; dB HL, decibels hearing level.

^a^ Factor scores of global cognition were developed using a validated latent variable modeling approach and standardized to the baseline, with higher scores indicating better cognitive function.

### Social Isolation

**Figure 2.**
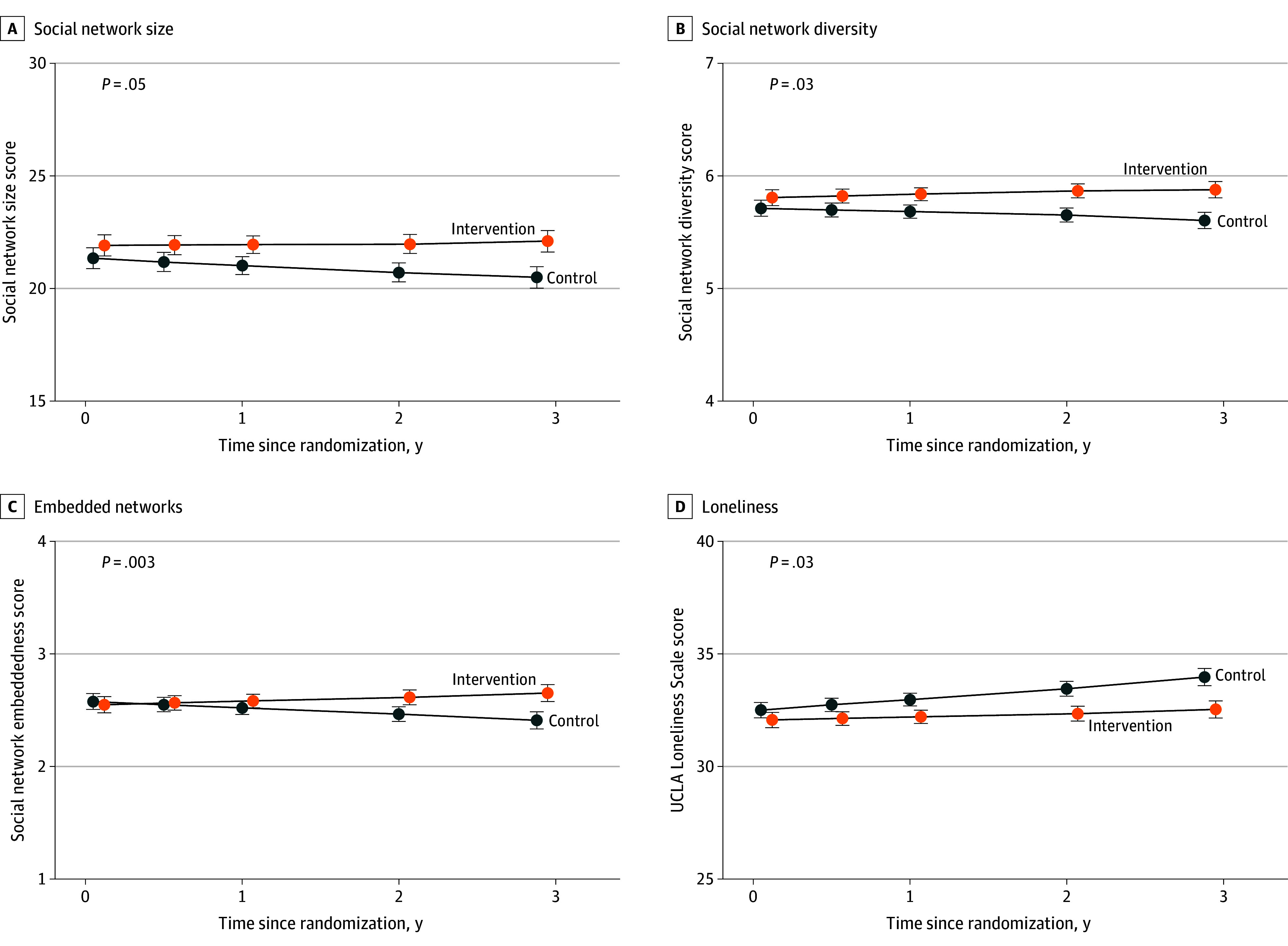
Trajectories and Pointwise Estimates of Social Network Characteristics and Loneliness by Randomly Assigned Treatment Among the Total Cohort of 977 Participants Y-axis values are social network characteristics and loneliness scores that were developed using the linear mixed-effect model. Parameter estimates and 95% CIs (indicated by whiskers) were calculated from a linear mixed-effects model that adjusted for baseline age, sex, education, field site, better-ear pure-tone average, speech-in-noise understanding, hearing handicap inventory for the elderly score, marital status, living alone, global cognition, Center for Epidemiologic Studies depression scale, antidepressant use, and whether the participant was part of a recruited spousal pair. Multiple imputation by chained equations was used to impute missing covariates.

**Figure 3.**
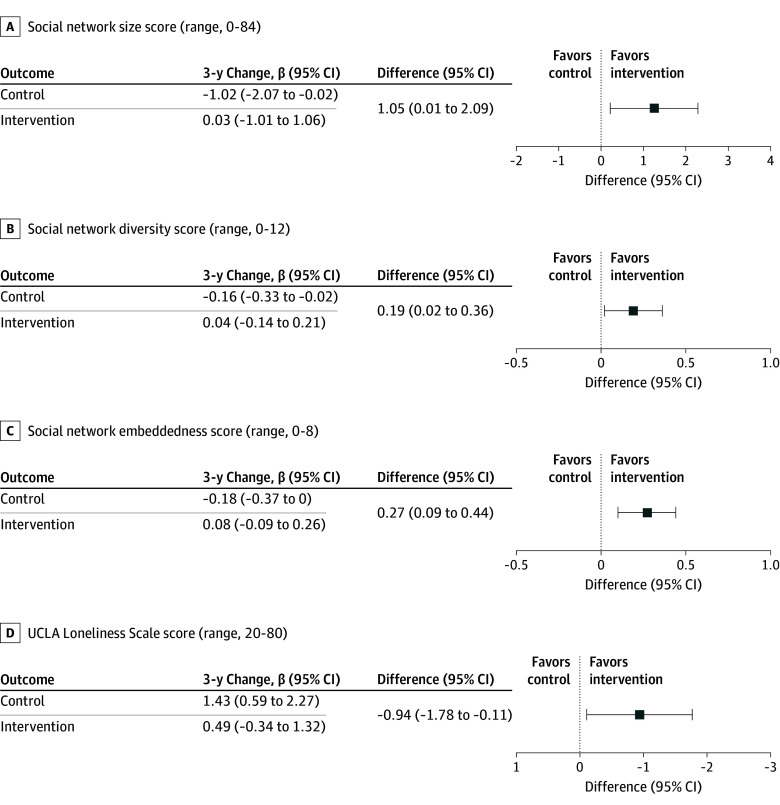
Covariate-Adjusted Analysis of 3-Year Change in Social Network Characteristics and Loneliness by Randomly Assigned Treatment Among the Total Cohort of 977 Participants Parameter estimates and 95% CIs were calculated from a linear mixed-effects model that adjusted for baseline age, sex, education, field site, better-ear pure-tone average, speech-in-noise understanding, hearing handicap inventory for the elderly score, marital status, living alone, global cognition, Center for Epidemiologic Studies depression scale, antidepressant use, whether the participant was part of a recruited spousal pair, and effects of the COVID-19 pandemic. Missing baseline covariate and missing social isolation and loneliness scores (measured at baseline and subsequent follow-up assessments during 3-year follow-up) were imputed using multiple imputation by chained equations. Postdeath assessments were excluded from imputation. Social isolation and loneliness scores were imputed (20 sets of imputations and 100 burn-in period interactions) separately and included all covariates from the fully adjusted model, as well as age (squared); interaction terms between age, race, and sex; time from baseline; and a 3-way interaction between time, intervention group, and recruitment source. Future social isolation and loneliness scores were excluded from the imputation model.

At baseline, mean (SD) social network size was 22.3 (10.2) in the health education control and 22.6 (11.1) in the hearing intervention. At year 3, mean (SD) social network size declined in the health education control (19.8 [10.2]) and hearing intervention (21.3 [11.0]); greater attrition in social network size was observed in the health education control (Figure 2). A similar pattern was observed for social network diversity and embeddedness (Figure 2). In covariate-adjusted linear mixed-effect models, hearing intervention was associated with reduced shrinkage in social network size during the 3-year study period (intervention, 0.03; 95% CI, −1.01 to 1.06; control, −1.02; 95% CI, −2.07 to −0.02; difference, 1.05; 95% CI, 0.01-2.09), corresponding to retention of a mean of 1 additional person in hearing intervention participants’ social network over 3 years compared with the health education control (Figure 2 and Figure 3). Similarly, hearing intervention was also associated with reduced shrinkage in social network diversity (intervention, 0.04; 95% CI, −0.14 to 0.21; control, −0.16; 95% CI, −0.33 to −0.02; difference, 0.19; 95% CI, 0.02-0.36) and social network embeddedness (intervention, 0.08; 95% CI, −0.09 to 0.26; control, −0.18; 95% CI, −0.37 to −0.00; difference, 0.27; 95% CI, 0.09-0.44) (Figure 2 and Figure 3).

### Loneliness

At baseline, mean (SD) UCLA loneliness scores were 32.7 (8.6) and 32.8 (8.4) among the health education control and hearing intervention groups, respectively. At year 3, mean (SD) scores slightly worsened to 33.5 (8.8) among the health education control while slightly improving to 32.3 (8.9) among the hearing intervention group. Adjusted linear mixed effects modeled demonstrated that hearing intervention reduced increases in loneliness during the 3-year study period (intervention, 0.49; 95% CI, −0.34 to 1.32; control, 1.43; 95% CI, 0.59-2.27; difference, −0.94; 95% CI, −1.78 to −0.11) (Figure 2 and Figure 3).

### Sensitivity Analyses

**Figure 4.**
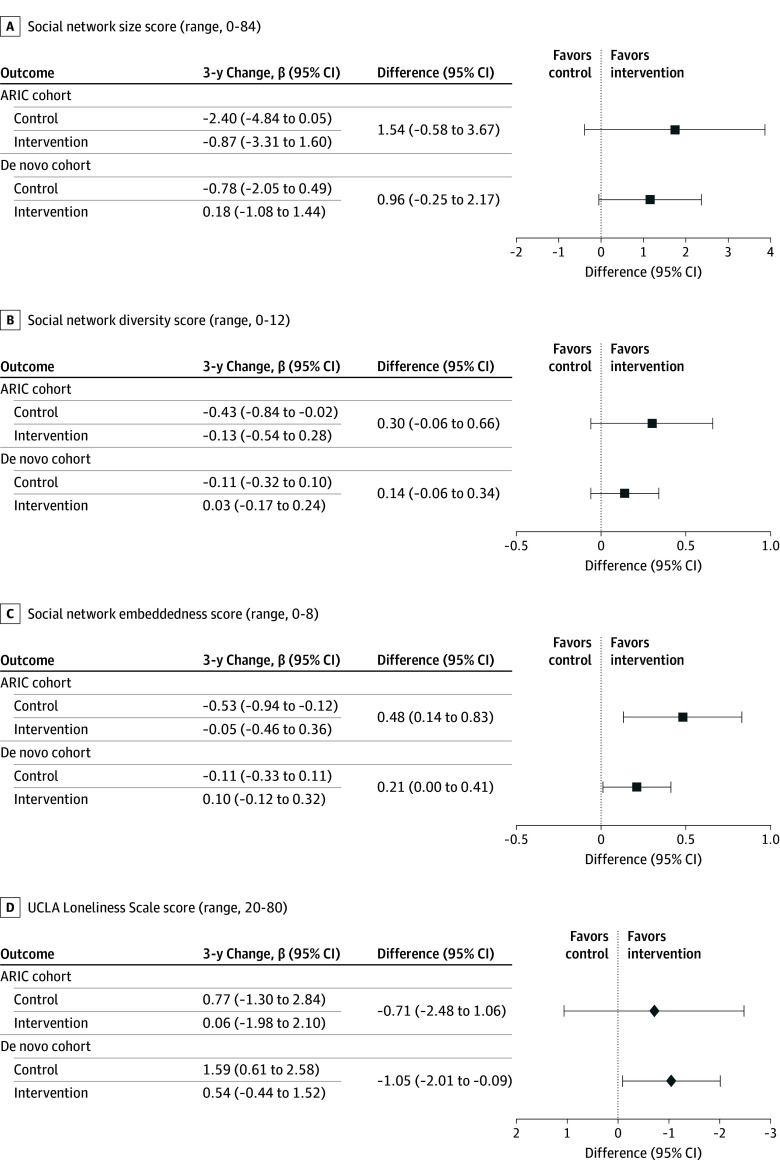
Covariate-Adjusted Analysis of 3-Year Changes in Social Network Characteristics and Loneliness by Randomly Assigned Treatment Stratified by Recruitment Source Parameter estimates and 95% CIs were calculated from a linear mixed-effects model that adjusted for baseline age, sex, education, field site, better-ear pure-tone average, speech-in-noise understanding, hearing handicap inventory for the elderly score, marital status, living alone, global cognition, Center for Epidemiologic Studies depression scale, antidepressant use, whether the participant was part of a recruited spousal pair, and effects of the COVID-19 pandemic. Missing baseline covariate and missing social isolation and loneliness scores (measured at baseline and subsequent follow-up assessments during 3-year follow-up) were imputed using multiple imputation by chained equations. Postdeath assessments were excluded from imputation. Social isolation and loneliness scores were imputed (20 sets of imputations and 100 burn-in period interactions) separately and included all covariates from the fully adjusted model, as well as age (squared); interaction terms between age, race, and sex; time from baseline; and a 3-way interaction between time, intervention group, and recruitment source. Future social isolation and loneliness scores were excluded from the imputation model. ARIC indicates Atherosclerosis Risk in Communities.

In analyses stratified by recruitment source, the effect of hearing intervention on social isolation and loneliness was consistent with the full population but with wider confidence intervals for some outcomes (Figure 4). Exclusion of covariates adjusting for the potential effect of nonpharmaceutical interventions associated with the COVID-19 pandemic, including physical distancing and lockdowns, did not change inferences across the models (eFigure 2 in Supplement 3). Per protocol analyses results were not substantially different from intention-to-treat analyses (eFigure 3 in Supplement 3). Complier average causal effect analyses supported the main findings, with slightly more pronounced effects for social network size and social network diversity and slightly attenuated effects for loneliness in the total cohort (eFigure 4 in Supplement 3). Stratified results by sex resulted in generally statistically insignificant results (eFigures 5 and 6 in Supplement 3).

## Discussion

In this secondary analysis of the ACHIEVE study, a hearing intervention had a positive effect across multiple distinct but related measures of social isolation and loneliness. The hearing intervention was associated with retaining a mean of 1 additional person in a participant’s social network over 3 years.

Our findings from a large, multisite randomized trial indicating a potential protective effect of hearing intervention on social isolation and loneliness measures over 3 years were consistent with previous observational studies but addressed some limitations (eg, residual confounding, limited characterization of the hearing intervention, and lack of a control group). In addition, our study added quantifiable social network measures to earlier work that was largely characterized by the use of subjective measures of perceived social engagement and loneliness. Among the few intervention studies on social and loneliness measures, Weinstein et al^13^ reported reductions from baseline De Jong Gierveld Loneliness Scale scores at 4 to 6 weeks following hearing aid intervention among 40 adults with hearing loss.^9^ However, Applebaum et al^27^ found no change from baseline UCLA loneliness scores at 6 months, 1 year, and 5 years among a clinical convenience sample of 64.^16^ Each of these studies included recruitment through a clinical setting that would likely result in participants with more significant perceived limitations from hearing loss or a perceived desire for hearing aids as opposed to the current study, which presents trial data on participants recruited from local communities based on their potential to benefit from hearing care irrespective of the degree of the perceived effect of hearing loss. The difference in populations could explain any differences in results, as clinical populations may be subject to selection bias and confounding by indication. Another major difference from the broader literature was that the current study included an active control (10 Keys) to balance the effects of time spent with a health professional. The active control may have biased results toward a null finding, as an improved healthy lifestyle (eg, managing blood pressure and increased physical activity) could also result in increased social contact.

Most published interventions for social isolation and loneliness among older adults have focused on counseling, skill-building sessions for social behaviors, enhancing opportunities for social interactions (eg, structured events or providing communications technology to improve access to others), and social prescribing.^1,2^ Relatively fewer studies have assessed the effectiveness of more distal interventions, such as exercise and music therapy.^15^ A pooled meta-analysis found mostly null effects of various interventions, with exceptions for music therapy in community settings, animal therapy, and technology (videoconferencing access) in long-term care settings, and multimodal interventions in both settings (albeit effect sizes were relatively small across all studies).^15^ Findings from this secondary analysis of the ACHIEVE study suggest that hearing intervention should also be routinely considered alongside other interventions to mitigate social isolation and loneliness, particularly given the high prevalence of hearing loss among older adults. Many previous interventions have focused on growing social networks to relatively heterogenous effects.^1,2,15^ An implication from these findings is that interventions focusing on prevention of further erosion of social networks could prove valuable additions to research and clinical recommendations.

Our results demonstrated positive effects of hearing intervention in the full cohort while stratified results by recruitment source were of similar magnitude and direction but with slightly wider confidence intervals, possibly related to smaller sample sizes. In contrast, the effect of hearing intervention on 3-year cognitive decline in the ACHIEVE study reported a null effect in the total cohort, yet prespecified sensitivity analyses reveled a strong effect in ARIC participants but no effect among de novo participants, among whom we observed a nearly 3-fold slower rate of cognitive change among controls relative to ARIC controls. A potential explanation is the healthy volunteer effect in the self-selected de novo cohort who responded to advertisements while the ARIC cohort represented individuals randomly sampled from the community when initially recruited into ARIC. Given the potential mediating role of social isolation in the association between hearing inventions and cognitive impairment, these findings could hint at possible positive effects of hearing intervention on reducing cognitive decline in the de novo cohort that may take longer than 3 years to observe. This long-term follow-up of the entire ACHIEVE cohort to 6 years is presently underway (NCT05532657).

### Limitations

Participants and study staff were not masked to intervention assignment, which may have influenced how participants responded to questions regarding social network characteristics and loneliness. Findings from this study also reflected a secondary analysis of a prespecified exploratory outcome of the ACHIEVE study rather than a direct primary or secondary outcome. As such, the study was not specifically designed or powered to investigate the effects of hearing intervention on social isolation and loneliness, and findings should be considered hypothesis-generating rather than hypothesis-testing. Future studies in this dataset should explore mediating and synergist effects between hearing intervention and outcomes. The COVID-19 pandemic may have affected the outcome, and the outcome measure instruments were not designed for phone-based administration. However, sensitivity analyses including calendar-time of the COVID-19 pandemic did not affect results and the baseline and final (year 3) end points were conducted in person. Additionally, the interpretation of the magnitude of our findings was limited by the scarcity of comparable studies. The lack of similar interventional research on social network scales and loneliness makes it challenging to contextualize the clinical significance of the observed effects. Lastly, the selective and healthy nature of the trial population and efficacy design may have limited generalizability. While the analysis of the ARIC population, which was closer to the general sociodemographic and health variables of the community, may have yielded some hints as to generalizability, trial transportability and further effectiveness studies are required to understand how hearing interventions could affect social and loneliness measures in the clinical setting.

The observed differences in the current study represent statistical differences, not necessarily clinically meaningful differences, among a general population of healthy, community-dwelling older adults with hearing loss. On the UCLA Loneliness Scale, a well-studied instrument considered valid and reliable, the average treatment effect observed would not constitute a change on the scales categorizations of loneliness.^1,26^ The Cohen Social Network Index, a relatively less well-studied instrument, does not have established clinically meaningful differences or associated categories. The main finding of social network size did not consider differences between contacts (eg, spouse vs colleague), and the 2-week time period could have been subject to recall bias.^1,2,6^ Moreover, as a secondary analysis, no consideration was given to specific levels of social isolation or loneliness as part of the inclusion criteria, so it is unknown whether the hearing intervention would improve loneliness measures or prevent social isolation among older adults with a higher baseline degree of loneliness or isolation. Given these constraints and the limited generalizability and efficacy design previously noted, the results should be interpreted cautiously. However, small statistical differences in social isolation and loneliness measures are associated with differences in health outcomes in observational data,^1,2,3,4,5,6,7,8,9,10,11^ rendering it plausible that the observed small statistical differences could result in overall health benefits at the population level.

## Conclusions

The results of this prespecified analysis of a randomized clinical trial characterize the potential effects of hearing intervention on reducing social isolation and loneliness in the ACHIEVE study, potentially contributing to the growing body of evidence suggesting that hearing intervention positively affects multiple areas of health, including cognition and communicative function.^16,28^ Our findings support recent initiatives from the Office of the Surgeon General^2^ of the US and National Academies^1^ in identifying interventions to promote social connection for improved health. Given the high prevalence of hearing loss^12^ among older adults and already established delivery models,^1^ hearing intervention represents a public health target for population-level reductions in social isolation and loneliness. Recent actions from the US Food and Drug Administration for an over-the-counter hearing aid^29^ category are a step in the right direction in improving access to hearing technologies. Additional efforts to incorporate coverage for hearing care and audiological support services as offered in the ACHIEVE study into Medicare benefits may further help improve access and affordability of hearing care for older adults.

## Notes

Supplement 1. Trial protocol

Supplement 2. Statistical analysis plan

Supplement 3. eTable 1. Demographic and Clinical Characteristics at Baseline of ACHIEVE Participants Stratified by Randomly Assigned Treatment and Recruitment Source (N=977) eTable 2. Social Network and Loneliness Characteristics of ACHIEVE Participants Stratified by Randomly Assigned Treatment and Recruitment Source (N=977) eTable 3. Social Network and Loneliness Characteristics of ACHIEVE Participants Stratified by Randomly Assigned Treatment and Recruitment Source (N=977) eFigure 1. Trial Profile eFigure 2. Sensitivity Analysis of 3-Year Change in Social Network Characteristics and Loneliness by Randomly Assigned Treatment among the Total Cohort and Stratified by Recruitment Source, Analysis not adjusted for COVID-19 (N=977) eFigure 3. Sensitivity Analysis of 3-Year Change in Social Network Characteristics and Loneliness by Randomly Assigned Treatment among the Total Cohort and Stratified by Recruitment Source, Per Protocol Analysis (N=824) eFigure 4. Sensitivity Analysis of 3-Year Change in Social Network Characteristics and Loneliness by Randomly Assigned Treatment among the Total Cohort and Stratified by Recruitment Source, Complier Average Causal Effect Analysis (N=977) eFigure 5. Sensitivity Analysis of 3-Year Change in Social Network Characteristics and Loneliness by Randomly Assigned Treatment among Female Participants (N=454) eFigure 6. Sensitivity Analysis of 3-Year Change in Social Network Characteristics and Loneliness by Randomly Assigned Treatment among Male Participants (N=523)

Supplement 4. Nonauthor collaborators

Supplement 5. Data sharing statement

## References

[1] National Academies of Sciences, Engineering, and Medicine. Social Isolation and Loneliness in Older Adults: Opportunities for the Health Care System. National Academies Press; 2020. 32510896

[2] Office of the Surgeon General. Our Epidemic of Loneliness and Isolation: The U.S. Surgeon General’s Advisory on the Healing Effects of Social Connection and Community. US Department of Health and Human Services; 2023. 37792968

[3] Cudjoe TKM, Roth DL, Szanton SL, Wolff JL, Boyd CM, Thorpe RJ. The epidemiology of social isolation: national health and aging trends study. J Gerontol B Psychol Sci Soc Sci. 2020;75(1):107-113. doi:10.1093/geronb/gby037 29590462 PMC7179802

[4] Shen C, Rolls ET, Cheng W, . Associations of social isolation and loneliness with later dementia. Neurology. 2022;99(2):e164-e175. doi:10.1212/WNL.0000000000200583 35676089

[5] Berkman LF, Glass T, Brissette I, Seeman TE. From social integration to health: Durkheim in the new millennium. Soc Sci Med. 2000;51(6):843-857. doi:10.1016/S0277-9536(00)00065-4 10972429

[6] Cohen S, Doyle WJ, Skoner DP, Rabin BS, Gwaltney JM Jr. Social ties and susceptibility to the common cold. JAMA. 1997;277(24):1940-1944. doi:10.1001/jama.1997.03540480040036 9200634

[7] Hawton A, Green C, Dickens AP, . The impact of social isolation on the health status and health-related quality of life of older people. Qual Life Res. 2011;20(1):57-67. doi:10.1007/s11136-010-9717-2 20658322

[8] Holt-Lunstad J, Smith TB, Layton JB. Social relationships and mortality risk: a meta-analytic review. PLoS Med. 2010;7(7):e1000316. doi:10.1371/journal.pmed.1000316 20668659 PMC2910600

[9] Huang AR, Roth DL, Cidav T, . Social isolation and 9-year dementia risk in community-dwelling Medicare beneficiaries in the United States. J Am Geriatr Soc. 2023;71(3):765-773. doi:10.1111/jgs.18140 36628523 PMC10023331

[10] Pomeroy ML, Cudjoe TKM, Cuellar AE, . Association of social isolation with hospitalization and nursing home entry among community-dwelling older adults. JAMA Intern Med. 2023;183(9):955-962. doi:10.1001/jamainternmed.2023.3064 37486647 PMC10366946

[11] Flowers L, Houser A, Noel-Miller C, . Medicare spends more on socially isolated older adults. Insight Issues. 2017;125:1119-1143. doi:10.26419/ppi.00016.001

[12] Reed NS, Garcia-Morales EE, Myers C, . Prevalence of hearing loss and hearing aid use among US Medicare beneficiaries aged 71 years and older. JAMA Netw Open. 2023;6(7):e2326320. doi:10.1001/jamanetworkopen.2023.26320 37505496 PMC10383002

[13] Weinstein BE, Sirow LW, Moser S. Relating hearing aid use to social and emotional loneliness in older adults. Am J Audiol. 2016;25(1):54-61. doi:10.1044/2015_AJA-15-0055 26999406

[14] Cacioppo S, Grippo AJ, London S, Goossens L, Cacioppo JT. Loneliness: clinical import and interventions. Perspect Psychol Sci. 2015;10(2):238-249. doi:10.1177/1745691615570616 25866548 PMC4391342

[15] Hoang P, King JA, Moore S, . Interventions associated with reduced loneliness and social isolation in older adults: a systematic review and meta-analysis. JAMA Netw Open. 2022;5(10):e2236676. doi:10.1001/jamanetworkopen.2022.36676 36251294 PMC9577679

[16] Lin FR, Pike JR, Albert MS, ; ACHIEVE Collaborative Research Group. Hearing intervention versus health education control to reduce cognitive decline in older adults with hearing loss in the USA (ACHIEVE): a multicentre, randomised controlled trial. Lancet. 2023;402(10404):786-797. doi:10.1016/S0140-6736(23)01406-X 37478886 PMC10529382

[17] Reed NS, Gravens-Mueller L, Huang AR, ; ACHIEVE Collaborative Research Group. Recruitment and baseline data of the Aging and Cognitive Health Evaluation in Elders (ACHIEVE) study: A randomized trial of a hearing loss intervention for reducing cognitive decline. Alzheimers Dement (N Y). 2024;10(1):e12453. doi:10.1002/trc2.12453 38356470 PMC10865776

[18] Deal JA, Goman AM, Albert MS, . Hearing treatment for reducing cognitive decline: design and methods of the Aging and Cognitive Health Evaluation in Elders randomized controlled trial. Alzheimers Dement (N Y). 2018;4(1):499-507. doi:10.1016/j.trci.2018.08.007 30364572 PMC6197326

[19] Sanchez VA, Arnold ML, Betz JF, ; ACHIEVE Collaborative Study. Description of the baseline audiologic characteristics of the participants enrolled in the Aging and Cognitive Health Evaluation in Elders study. Am J Audiol. 2024;33(1):1-17. doi:10.1044/2023_AJA-23-00066 38166200 PMC11001432

[20] Sanchez VA, Arnold ML, Reed NS, . The hearing intervention for the Aging and Cognitive Health Evaluation in Elders randomized control trial: manualization and feasibility study. Ear Hear. 2020;41(5):1333-1348. doi:10.1097/AUD.0000000000000858 32251012 PMC10436703

[21] Arnold ML, Haley W, Lin FR, . Development, assessment, and monitoring of audiologic treatment fidelity in the aging and cognitive health evaluation in elders (ACHIEVE) randomised controlled trial. Int J Audiol. 2022;61(9):720-730. doi:10.1080/14992027.2021.1973126 34533430 PMC11992692

[22] Deal JA, Albert MS, Arnold M, . A randomized feasibility pilot trial of hearing treatment for reducing cognitive decline: Results from the Aging and Cognitive Health Evaluation in Elders pilot study. Alzheimers Dement (N Y). 2017;3(3):410-415. doi:10.1016/j.trci.2017.06.003 29067347 PMC5651440

[23] Newman AB, Bayles CM, Milas CN, . The 10 keys to healthy aging: findings from an innovative prevention program in the community. J Aging Health. 2010;22(5):547-566. doi:10.1177/0898264310363772 20495156 PMC4896138

[24] Venditti EM, Zgibor JC, Vander Bilt J, . Mobility and Vitality Lifestyle Program (MOVE UP): a community health worker intervention for older adults with obesity to improve weight, health, and physical function. Innov Aging. 2018;2(2):igy012. doi:10.1093/geroni/igy012 30480135 PMC6176958

[25] Morone NE, Greco CM, Moore CG, . A mind-body program for older adults with chronic low back pain: a randomized clinical trial. JAMA Intern Med. 2016;176(3):329-337. doi:10.1001/jamainternmed.2015.8033 26903081 PMC6361386

[26] Russell D, Peplau LA, Cutrona CE. The revised UCLA Loneliness Scale: concurrent and discriminant validity evidence. J Pers Soc Psychol. 1980;39(3):472-480. doi:10.1037/0022-3514.39.3.472 7431205

[27] Applebaum J, Hoyer M, Betz J, Lin FR, Goman AM. Long-term subjective loneliness in adults after hearing loss treatment. Int J Audiol. 2019;58(8):464-467. doi:10.1080/14992027.2019.1593523 30929531 PMC10436704

[28] Sanchez VA, Arnold ML, Garcia Morales EE, ; ACHIEVE Collaborative Study. Effect of hearing intervention on communicative function: a secondary analysis of the ACHIEVE randomized controlled trial. J Am Geriatr Soc. 2024;72(12):3784-3799. doi:10.1111/jgs.19185 39266468 PMC11637286

[29] Lin FR, Reed NS. Over-the-counter hearing aids: how we got here and necessary next steps. J Am Geriatr Soc. 2022;70(7):1954-1956. doi:10.1111/jgs.17842 35512226

