# Supplementary material for: Hearing Intervention, Social Isolation, and Loneliness: A Secondary Analysis of the ACHIEVE Randomized Clinical Trial
Source: JAMA Intern Med. 2025 May 12;185(7):797–806. doi: 10.1001/jamainternmed.2025.1140 (PMC12070280; doi:10.1001/jamainternmed.2025.1140)
Supplement: Supplement 2. — Statistical analysis plan [file jamainternmed-e251140-s002.pdf]

STATISTICAL ANALYSIS PLAN: ACHIEVE

PROTOCOL: Aging and Cognitive Health Evaluation in Elders (ACHIEVE) Randomized Trial

GRANT Number R01AG055426

NCT Number NCT03243422

SAP VERSION 5.2

SAP DATE: February 23, 2023

PROTOCOL DATE: November 9, 2021 (V.1.10)

SPONSOR: National Institute on Aging

PREPARED BY: Collaborative Studies Coordinating Center
Department of Biostatistics
University of North Carolina at Chapel Hill

AUTHORS: Will be listed in order of contribution as draft is prepared

REVISIONS HISTORY:

Version	Author	Date	Description
1	David Couper, Lisa Gravens-Mueller	9/4/20	Original SAP
2	David Couper, James Pike, Lisa Gravens-Mueller	4/15/21	Updated SAP due to COVID 19
3	David Couper, James Pike, Lisa Gravens-Mueller	8/20/21	Additional updated due to COVID 19
4	David Couper, James Pike, Lisa Gravens-Mueller	3/14/22	Details added to clarify analytic plan
5	David Couper, James Pike, Lisa Gravens-Mueller	4/27/22	Noted in section 7.5 that results from the analysis of secondary outcomes will be included in primary outcome manuscript
5.1	David Couper, James Pike, Lisa Gravens-Mueller	11/11/22	Minor Update: Clarified definition of secondary outcome and described sensitivity analyses.
5.2	David Couper, James Pike, Lisa Gravens-Mueller	2/23/23	Minor Update: Clarified use of Hochberg modification to the Bonferroni adjustment.

Changes from version 1.0 to 2.0

Affected section	Brief description of change	Brief rationale for change
4. Study Design	Added: Each participant will be followed for 36 months; the COVID-19 pandemic made in person visits impossible from mid-2020 to mid-2021 delaying the closeout 36 month visit for many participants.	Due to COVID-19
4. Study Design	Added: Four intervention visits occur every three weeks following randomization. Routine follow-up clinic visits occur every six months until month 36. The purpose is to gather measures of primary and secondary outcomes. Starting in April 2020, remote data collection of primary and secondary outcomes became necessary due to the coronavirus pandemic (see section 7.1 for description of alternative modes of data collection). In January 2021, due to the coronavirus pandemic, it was decided participants' final study visit will be a split visit that will comprise first a phone-based component AND later an in-person component (with final cognitive assessment and receipt of the other study intervention) that will be conducted once field site clinics reopen in the future.	Due to COVID-19
7.1 General Considerations	Removed the third mode of neurocognitive data collection (Video Conferencing). Updated the calibration analysis and noted that factor analysis will be performed using MPlus 8.6 or later	It was decided not to use the video conferencing for neurocognitive data collection during COVID-19
7.3 Primary Analysis	Updated to note if a nonlinear trend is observed, the model will be adapted to include time splines. Updated the covariates to sex, race-center, and education, Cohort type (ARIC vs De novo. Removed the word "ANCOVA" from all covariates included in the ANCOVA model	Due to COVID-19 neurocognitive collection will be over the phone until in-person visits can resume
7.4 Sensitivity Analyses of the Primary Outcome	Updated one of the sensitivity analyses to look at cognitive decline based on a categorized version of time (visit) rather than continuous time in years	Due to COVID-19 the amount of follow-up time will be longer than 3 years for some participants.

7.6 Exploratory Analyses of the Primary Outcome	Updated the Exploratory analysis looking at whether or not the intervention alter the trajectory of cognitive decline	Due to COVID-19
10. COVID-19	Added information regarding conducting the year 3 visit as a split visit.	Due to COVID-19

Changes from version 2.0 to 3.0

**Affected section**	**Brief description of change**	**Brief rationale for change**
7.1 General Considerations	Updated administration of the neurocognitive assessment to note in-person visits resumed in June 2021. Removed the paragraph on calibration.	Due to COVID-19
7.3 Primary Analysis	Noted that we will be using both the in-person and telephone based neurocognitive assessments.	Due to COVID-19
7.4 Sensitivity Analyses of the Primary Outcome	Added a new Sensitivity analysis "An analysis of a co-calibrated[10,11] global cognitive function factor scores in which the ten tests administered in-person and the six tests administered over the phone will be included in the confirmatory factor analysis model. Modeling constraints will be applied to scale the co-calibrated factor score to the same metric as the in-person factor score utilized in the primary analysis[21]. Mode of data collection (in-person, phone) will be included in the analytic model as a covariate."	Due to COVID-19
7.5 Key Secondary Outcomes	Clarified which secondary outcome would be consider the main and elaborated the definitions.	Due to COVID-19

Changes from version 3.0 to 4.0

**Affected section**	**Brief description of change**	**Brief rationale for change**
7.1, General Considerations and 7.3 Primary Analysis	Added threshold for dichotomization of hearing loss and removed the ambiguous term "stratum".	Clarified how hearing loss would be defined as a covariate in the primary analysis.
7.1 General Considerations 7.3 Primary Analysis	Removed phone-based cognitive scores and added race to the imputation model. Removed the sentence "The phone-based global cognitive function factor score will be included in the imputation model as an auxiliary variable."	A test of 35 separate models revealed that phone-based global cognitive function factor scores did not improve the performance of the imputation model. However, adding race as an auxiliary variable resulted in a small improvement.

7.3 Primary Analysis	For the primary analysis the time scale was specified as "years from the baseline."	Clarified the timeframe for cognitive decline.

Changes from version 4.0 to 5.0

Affected section	Brief description of change	Brief rationale for change
7.5 Secondary Outcomes	Clarified that results from the analysis of secondary outcomes will be included in the primary outcome manuscript	Specified the importance of examining intervention effects on both cognitive decline and adjudicated dementia.

Changes from version 5.0 to 5.1

Affected section	Brief description of change	Brief rationale for change
7.5 Secondary Outcomes	Clarified definition of secondary outcome and described sensitivity analyses.	Specified source of information for adjudicated MCI and dementia.

Changes from version 5.1 to 5.2

Affected section	Brief description of change	Brief rationale for change
7.7 Adjustment for Multiple Comparisons	Clarified use of Hochberg modification to the Bonferroni adjustment.	Specified that the Hochberg modification to the Bonferroni adjustment will be applied to five outcomes (1) decline in global cognitive function, (2) time to incident cognitive impairment, (3) decline in memory domain, (4), decline in executive function domain, and (5) decline in language domain.

<u>SAP Signatures</u>

DSMB Board and National Institute on Aging June 2, 2022

Principal Investigator – Frank Lin, MD, PhD May 16, 2022

Protocol Statistician – David Couper, PhD May 16, 2022

TABLE OF CONTENTS

1. Introduction 7
2. Background 7
3. Objectives 7
4. Study Design 7
   4.1 Inclusion Criteria 8
   4.2 Exclusion Criteria 9
5. Power 9
   5.1 Preliminary Analysis 9
   5.2 Evaluation of Assumptions 10
6. Analysis Populations 10
   6.1 Intent-to-Treat Population 10
   6.2 Per Protocol and Complier Population 11
7. Statistical Analysis 11
   7.1 General Considerations 11
   7.2 Primary Outcome 12
   7.3 Primary Analysis 13
   7.4 Sensitivity Analyses of the Primary Outcome 13
   7.5 Secondary Outcomes 14
   7.6 Exploratory Analyses of the Primary Outcome 14
   7.7 Adjustment for Multiple Comparisons 15
   7.8 Additional Outcomes 15
8. Global cognitive function factor score 15
9. COVID-19 Considerations 16
10. Software and Statistical Programming 16
11. References 16

LIST OF ABBREVIATIONS

ACTIVE	Advanced Cognitive Training for Independent and Vital Elderly study
AD	Alzheimer's Disease
APOE	Apolipoprotein E
ARIC	Atherosclerosis Risk in Communities
CDR	Clinical Dementia Rating
dB	decibels
DSMB	Data and Safety Monitoring Board
HealthABC	The Health, Aging, and Body Composition Study
HRQL	Health-Related Quality of Life
ITT	Intention-to-treat
MCI	Mild Cognitive Impairment
MMSE	Mini-Mental State Exam
SD	Standard deviation

1. Introduction

ACHIEVE is a randomized controlled trial. Approximately 850 participants will be randomly assigned to one of two conditions and followed for three years: hearing intervention with hearing aids or successful aging intervention. Participants will be community-dwelling adults aged 70-84 years with audiometric hearing impairment defined as a four-frequency (0.5, 1, 2, 4 kHz) pure-tone average threshold in the better-hearing ear of ≥30 decibels (dB) and <70dB. This document gives details of the planned statistical analyses.

2. Background

Novel approaches for reducing cognitive decline in older adults are needed given the aging of the population and the personal, socioeconomic, and public health implications of cognitive impairment and dementia in older adults. Epidemiologic data now strongly suggest that age-related peripheral hearing loss in older adults is independently associated with accelerated cognitive decline and incident dementia[1-4]. Mechanistic pathways that could underlie this observed association include the effects of poor audition and distorted peripheral encoding of sound on cognitive load, brain structure, and/or reduced social engagement. These pathways may be amenable to comprehensive hearing rehabilitative treatment consisting of the use of hearing assistive technologies (hearing aids, other integrated hearing assistive devices) and rehabilitative training. To date, however, there has never been a randomized trial that has investigated whether hearing loss treatment could reduce cognitive and other functional declines in older adults.

3. Objectives

The main objective of ACHIEVE is to determine the effect of a hearing intervention versus a successful aging control intervention on the primary outcome of global cognitive decline in 70-84 year-old well-functioning and cognitively-normal adults with hearing loss. An important secondary outcome is time until cognitive impairment (defined in section 7.5). Additional secondary outcomes include decline in the cognitive domains of memory, executive function, and language. Changes in social and physical function, physical activity, and health-related quality of life (HRQL) will be examined in exploratory analyses.

Additional exploratory objectives in ACHIEVE include: 1) to investigate whether the hearing intervention alters within-individual pre- to post-intervention trajectories of cognitive decline in the subgroup of participants recruited from the Atherosclerosis Risk in Communities (ARIC) cohort who have been followed for ~30 years prior to enrollment in the ACHIEVE trial; and 2) to evaluate the effect of hearing intervention on rates of cognitive decline in individuals with different demographics, biomarkers, and Alzheimer’s disease risk factors.

4. Study Design

ACHIEVE is a randomized, open-label trial comparing a hearing intervention versus a successful aging control intervention. The original plan for enrollment was to recruit 850 participants with approximately 425 in each condition. The final enrollment was expanded to 977 participants following a sample size assumption analysis (see section 5.2) conducted in June 2019. Enrollment began in November 2017. Each participant will be followed for 36 months; the COVID-19 pandemic made in-person visits impossible from mid-2020 to mid-2021 delaying the closeout 36 month visit for many participants.

Participants in the hearing intervention condition are fitted with a hearing aid, instructed to be worn on a daily basis for study duration, and attend four 1-hour intervention sessions spaced over the 2-3 months post-randomization. Re-instruction in use of devices and hearing rehabilitative strategies will be provided every 6 months.

The successful aging control intervention consists of four 1-hour intervention sessions spaced over the 2-3 months post-randomization focusing on participant-selected topics from the 10 Keys™ to Healthy Aging program and upper body stretching. Additional sessions will be provided every 6 months.

When spouses or cohabitating partners are both eligible and randomized together, they will be randomized as a unit with, arbitrarily, the first spouse/partner of the pair to be selected according to the random assignment procedure and the second spouse/partner of the pair receiving the same assignment. Spouse/partner pairs will be randomized in spouse/partner-pair specific permuted order blocks of varying sizes within strata defined by participant status (at least one spouse/partner pair in ARIC or both non-ARIC participants) and by field site.

Pre-screening will determine if an individual meets the inclusion criteria. The screening visit is used to consent participants and to determine if an individual meets audiometric, vision, and cognitive inclusion criteria. During the baseline visit assessments measures of primary and secondary outcomes will be collected.

Four intervention visits occur every three weeks following randomization. Routine follow-up clinic visits occur every six months until month 36. The purpose is to gather measures of primary and secondary outcomes. Starting in April 2020, remote data collection of primary and secondary outcomes became necessary due to the coronavirus pandemic (see section 7.1 for description of alternative modes of data collection). In January 2021, it was decided participants' final study visit will be a split visit that will comprise a phone-based component AND a subsequent in-person component (with final cognitive assessment and receipt of the other study intervention) that will be conducted once field site clinics reopen following closures prompted by the coronavirus pandemic.

4.1 Inclusion Criteria

To be eligible for the study, participants must meet all of the following criteria:

- Age 70-84 years at the time of randomization. This age range will allow recruitment of participants who are at risk for cognitive decline but who may also be expected to survive for the duration of the study.
- Community-dwelling.
- Fluent English-speaker.
- Residency. Participants must plan to reside in the local area for the study duration.
- Audiometric hearing impairment. Participants must have adult-onset hearing impairment with a four-frequency pure tone average (0.5, 1, 2, 4 kHz) in the better-hearing ear of ≥ 30 decibels and <70dB. This level of hearing impairment is the level at which individuals would be most likely to benefit from the use of conventional amplification devices such as hearing aids.
- Word recognition in quiet score ≥60% in better ear. A word recognition in quiet score <60% suggests hearing impairment that is too severe to benefit from conventional amplification devices such as hearing aids.
- Mini-Mental State Exam (MMSE) score ≥ 23 for individuals with high-school degree or less; MMSE score ≥ 25 for individuals with some college or more; Participants must be at risk for cognitive decline in the range quantified well by neurocognitive testing, and so must be free from more substantial cognitive impairment at baseline.

Participants must be willing and able to consent to participate in the study, be willing to be randomized to either the hearing intervention or to the successful aging control intervention and be willing to commit to adhere to the protocol for the duration of the study.

4.2 Exclusion Criteria

Potential candidates for enrollment who meet one or more of the following criteria are excluded from participation in the study:

- Self-reported disability in ≥ 2 or more Activities of Daily Living.
- Any self-reported hearing aid use in the past year. Participants will be randomized to hearing intervention or successful aging control intervention and, therefore, participants cannot already be receiving treatment for their hearing loss.
- ARIC participants only: Diagnosis of adjudicated dementia based on a previous ARIC visit or participant required a proxy to assist with completing informed consent and responding to questions at ARIC visit 6 or 7.
- Vision impairment (worse than 20/40 on MN Near Vision Card). Participants who cannot see (with correction) well enough to complete the neurocognitive assessment are excluded.
- Medical contraindication to use of hearing aids (e.g., draining ear). Because hearing aids will be the primary device used in the hearing intervention, participants with medical contraindications to hearing aid use are excluded.
- Conductive hearing impairment as determined by a difference in air audiometry and bone audiometry ("air-bone gap") greater than 15 dB in 2 or more contiguous frequencies in both ears. Because the impact of a conductive (versus a sensorineural) hearing loss on cognitive functioning may potentially differ and programming for hearing aids differs for conductive hearing loss, participants with permanent conductive hearing loss are excluded from the study. Participants with an air-bone gap due to fluid in the ears or other resolvable medical issue may be enrolled in the study following successful medical resolution of the cause of the air-bone gap.
- Unwilling to wear hearing aids on a regular (i.e., daily or near daily) basis.
- No participants are excluded based on race or sex.

Temporary Exclusion Criteria: Participants determined to have a conductive hearing impairment as measured by a difference in air audiometry and bone audiometry ("air-bone gap") greater than 15 dB in 2 or more contiguous frequencies in both ears are excluded from the study and referred for medical follow-up. Should the cause of the air-bone gap be determined to be due to a temporary or treatable medical issue (e.g., fluid or wax in the ears), participants may be enrolled following resolution of the issue (i.e., air-bone gap). Participants with asymmetrical sensorineural hearing (≥20 dB at 2 adjacent frequencies or ≥ 10 dB at 3 adjacent frequencies) or with other symptoms/signs concerning for a retrocochlear etiology based on the assessment of the audiologist will also not be eligible to participate until a medical clearance/evaluation is obtained.

5. Power

5.1 Preliminary Analysis

Data from the ARIC cohort, the Advanced Cognitive Training for Independent and Vital Elderly (ACTIVE) randomized trial, and the Health Aging and Body Composition (HealthABC) study were utilized to estimate that in the absence of an intervention a factor score (described in section 8.0) of global cognitive function would decline at a rate of 0.08 standard deviations (SDs) per year with a variability of 0.09 SDs. These estimates of the slope and variance were translated into expected values at Year 3. Power was conservatively estimated using a simplified t-test approach, while recognizing that greater power would be provided in the analysis of the primary outcome of cognitive decline through the application of repeated measures mixed effects models (described in section 7.3). The original sample size for ACHIEVE was 850, which provided greater than 90% power to detect a 35% difference in annual cognitive decline (Intervention: -0.052, Control: -0.08) with a 2-sided test and $p < 0.05$.

Attrition due to withdrawal or mortality was estimated at 10% per year, corresponding to 27.1% over 3 years. This is a conservative estimate given that *total* attrition in ACHIEVE is 6.7% as of October 6th, 2021. The drop-in/drop-out rate was estimated at 15% over 3 years. This rate reflects participants in the successful aging control condition who begin to wear hearing aids (drop-in) and participants in the hearing intervention condition who stop wearing hearing aids (drop-out). As of October 6th, 2021 there are 46 participants in the control condition who are currently using hearing aids (9.4% drop-in) and 8 participants in the intervention condition who are not currently using hearing aids (1.6% drop-out). After accounting for a 15% drop-in/drop-out rate, the difference at Year 3 was reduced to 29% (Intervention: -0.057, Control: -0.08). With an attrition rate of 10% per year, the initial sample size for ACHIEVE provided greater than 80% power to detect a difference utilizing a 2-sided t-test and $p < 0.05$.

For the secondary outcome of incident cognitive impairment, a dataset was constructed of participants in the ARIC cohort who had comparable characteristics to the individuals enrolled in ACHIEVE, did not exhibit signs of cognitive impairment at the baseline, and who were monitored over a period of 5 to 8 years. The observed rate was 50 cases of incident cognitive impairment per 1000 person-years. Assuming a 30% reduction in the hearing intervention condition and a 25% loss of person-time due to attrition, the proposed proportional-hazards model (described in section 7.5) would have 80% power to detect a hazard ratio of 0.54 when using a 2-sided test and $p < 0.05$.

5.2 Evaluation of Assumptions

An assumptions analysis took place in June 2019, prior to the closure of the initial recruitment period. The goal of the analysis was to evaluate a subset of the assumptions described above and assess the value of a possible increase in the sample size. Since ACHIEVE was not going to be stopped early for efficacy or futility, regardless of the interim results, and the interim analysis only investigated assumptions around attrition and drop-in/drop-out rates, not outcomes, the interim analysis did not lead to increased type I error rates. Moreover, it was important to determine the extra required sample size, if any, as soon as possible in order to promptly begin the second stage of recruitment and expand the sample while there were still active participants from the initial stage.

Both the attrition rate and the drop-in/drop-out rate were examined based on 1 year follow-up data from approximately 200 participants. The data suggested that the original assumptions made in the sample size calculations were reasonable. However, there was potential variability in the estimates since partial data were used in the analysis. Consequently, the DSMB recommended increasing the sample size up to 1000 participants. The final sample size is 977 participants. This increased sample provided additional power for all tests, including greater than 90% power to detect a difference in cognitive decline after accounting for the estimated attrition and drop-in/drop-out rate as well as 80% power to detect a hazard ratio of 0.60 in the proportional-hazards analysis of incident cognitive impairment.

6. Analysis Populations

6.1 Intent-to-Treat Population

Analyses will follow the intention-to-treat (ITT) principle in which subjects will be analyzed in the condition to which they were randomized, regardless of whether they received the assigned intervention. Primary analyses will be based on the ITT population, which includes all randomized subjects.

6.2 Per Protocol and Complier Population

A secondary analysis of the primary outcome will be completed for the per-protocol population, defined as a subset of the ITT population who completed the 2-3 months intervention period, had no hearing aid intervention drop-in for the control condition, and had no major protocol deviations. Major protocol deviations include violations in inclusion and exclusion criteria at enrollment and poor compliance with hearing aids for the hearing aid intervention condition, defined as subjects who discontinue hearing aid use. All major protocol deviations will be identified in a blinded fashion prior to database lock. Complier average causal effect analysis will also be conducted to mitigate bias present[5] in per protocol analyses.

7. Statistical Analysis

7.1 General Considerations

Study data will be monitored on an on-going basis by the ACHIEVE Data Coordinating Center (DCC). The DCC will send data clarification requests to the clinical sites for resolution while the study is on-going. Final cleaning and editing of the study database will be carried out after the final participant completes their last follow-up visit. Unblinding of treatment assignments will not be performed until the study data are cleaned, queries resolved, and database lock achieved. A permanent archive of the database will be maintained by the DCC. Randomization, stratified by ARIC study status, center, and hearing impairment level is completed within CDART, the DCC's data management system. Built-in algorithms first check for missing key items and eligibility criteria, reducing protocol violations associated with enrollment. Although the ACHIEVE hearing aid intervention is, by nature, un-masked, to minimize bias based on review of accumulating data by the project team, the ACHIEVE PI, co-investigators, and key project staff, except for key data coordinating center staff, will remain blinded to accumulating data. This will be done by applying a dummy ID onto the treatment-specific data forms as they are transferred from CDART to SAS for data cleaning and interim reporting.

This statistical analysis plan (SAP) was developed prior to review of the study dataset and under the assumption that all data were collected in adherence to the protocol and in accordance with good clinical research practices. As the data are analyzed, some deviation is anticipated (e.g., missing data; small sample sizes). In instances where these deviations would make the proposed analyses inappropriate, modifications to the analysis plan will be made and noted in the final report.

Consistent with best practices in clinical trials, we will assess the comparability of the randomized conditions with respect to known baseline confounders, such as hearing loss (pure tone average in the better hearing ear <40 db vs 40+ db), recruitment source (ARIC vs de novo), center, age, sex, education, and the presence of APOE ε4 alleles[6]. If, despite randomization, there is an imbalance between the treatment conditions we will adjust for relevant confounders as well as explore the use of a precise, locally efficient, augmented, simple estimator[7] which may offer greater precision.

At the outset of the study, an in-clinic neurocognitive assessment comprised of ten tests (see table 7.1) was administered. The assessment included the delayed word recall (exposure and recall)[8] logical memory test (I and II)[9], incidental learning test[10], word fluency test (letters F, A, and S)[11], animal naming test[11], Boston naming test[12], digit symbol substitution[9], trail making tests parts A and B[13], and the digit span backwards[9]. All ten tests were used to compute a global cognitive function factor score (described in section 8.0) for each participant[14] that was standardized to the baseline of the study.

Due to the outbreak of COVID-19 (see section 9.0 for details), a modified six-test phone-based neurocognitive assessment was administered beginning in July 2020. The assessment included the Consortium to Establish a Registry for Alzheimer's Disease (CERAD) word list (immediate and delayed)[15]

an abbreviated version of the word fluency test (letters F and A), the animal naming test, oral versions of trail making tests parts A and B, and the digit span backwards. The six tests were utilized to compute a phone-based global cognitive function factor score.

In-person administration of the original ten tests resumed in June 2021. All ACHIEVE participants will be invited to complete an in-person assessment even if the target date for the 36-month follow-up has passed. If a participant does not complete an in-person assessment, an in-person global cognitive function factor score will be imputed (see section 7.3).

Table 7.1. ACHIEVE Neurocognitive Test Battery Components

ACHIEVE in-clinic test order[1]	ACHIEVE telephone test order[1]
Ensuring Speech Understanding	Ensuring Speech Understanding for Telephone
Mini Mental State Exam (MMSE)	MMSE / CDR - Participant Hybrid for Telephone[2]
Delayed Word Recall - Exposure	CERAD Immediate[3]
Digit Symbol Substitution	Digit Span Backward
Delayed Word Recall	CERAD Delayed[3]
Incidental Learning	Oral Trails A[2]
Word Fluency (FAS)	Oral Trails B[2]
Animal Naming	Word Fluency (FA)
Logical Memory I	Animal Naming
Digit Span Backwards	
Trails A	
Trails B	
Boston Naming	
Logical Memory II	

[1]Test order changes across modes of administration to enable timing delays for memory tests.
[2]Modification applied to the in-clinic version of the neurocognitive test for telephone administration.
[3]Indicates neurocognitive test not included in the original 10-test battery.

All programs used in the statistical analysis of study data will be documented, tested, and archived. Archiving of statistical analyses at the DCC includes the original written specifications for the analyses, any subsequent modifications, the computer program file, and the log, list and other output files produced by the program. The DCC will undertake all the analyses of study data using SAS® version 9 or later. The factor analysis will be performed using Mplus 8.6 or later.

7.2 Primary Outcome

The primary outcome is cognitive decline, as measured by the change in a global cognitive function factor score. The factor score is derived from a confirmatory factor analysis model that identifies common covariation among all cognitive tests administered in-person. Factor scores are generated for each participant at each in-person assessment using a measure harmonization and item banking approach[16, 17]. Factor loadings and latent means for each neurocognitive test are estimated using data from the ACHIEVE baseline. The mean of the global cognitive function factor score at the baseline is set to 0 and the SD is set to 1. Fixed parameter estimates from the baseline model are then integrated into models that estimate the factor mean and SD at each subsequent assessment. This process ensures that changes over time represent changes in the underlying factor rather than changes in the scaling or meaning of the constructs. The assumption of longitudinal measurement invariance is supported by prior studies that utilized similar neurocognitive tests[18, 19]. A final fixed parameter model analyzes available data collected in-person and

computes a global cognitive function factor score for each participant at every assessment in which one or more neurocognitive tests were completed. A comparable process is used to generate factor scores for the cognitive domains of memory, executive functioning, and language.

7.3 Primary Analysis

We will examine cognitive decline within each condition using mixed effects models that account for the correlation among repeated measures as well as the correlation between spouses or cohabitating partners. If a linear trend appears reasonable, we will fit a model with a linear slope. If a nonlinear trend is observed, the model will be adapted to include time splines[20]. Continuous time in years from the baseline will be the time scale. An interaction term between treatment assignment and time will be used to test if rates of cognitive change differ by treatment assignment. Model fit will be assessed with residual plots and other statistics (Akaike Information Criterion, Bayesian Information Criterion, etc.). The primary analysis may include adjustments for the baseline hearing loss, ARIC vs de novo status, center, age, sex, education, and APOE ε4 alleles.

Missing cognitive factor scores among ACHIEVE participants will be generated utilizing multiple imputation by chained equations[21]. The number of imputations needed to generate valid parameter estimates will be determined by a two-stage analysis[22]. The imputation model will include (1) in-person cognitive factor scores, (2) MMSE and Six-item Screener scores, (3) adjudicated incident MCI or dementia, (4) race, (5) time variables indicating when a participant with missing data might have completed an assessment based on time from randomization to missed visit, and (6) all previously listed covariates. Interactions between variables in the imputation model will be tested and added as necessary. The imputation will be conducted in stages so that concurrent and past measurements, but not future measurements, inform the imputed values. The validity of the imputation model will be assessed by comparing observed values to imputed values among a 20% sample selected at random and a 20% sample selected based on the probability of missingness estimated from a logistic regression model.

The primary analysis will focus on cognitive factor scores imputed prior to death. An analysis comparing pre- and post-death cognitive factor scores will be performed using values generated from an imputation model in which death is included as an auxiliary variable. A similar procedure will be used to contrast pre- and post-dementia cognitive factor scores.

7.4 Sensitivity Analyses of the Primary Outcome

Additional analyses of the primary outcome may include, but are not limited to:

(a) An analysis of global cognitive function in which data from in-person cognitive evaluations will be stratified by condition before a factor score is computed for each participant. The factor scores generated will be standardized using the condition-specific baseline mean and SD.
(b) An analysis of a co-calibrated[23,24] global cognitive function factor score in which the ten tests administered in-person and the six tests administered over the phone will be included in the confirmatory factor analysis model. Modeling constraints will be applied to scale the co-calibrated factor score to the same metric as the in-person factor score utilized in the primary analysis[25]. Mode of data collection (in-person, phone) will be included in the analytic model as a covariate.
(c) Use of full-information maximum likelihood to account for missing data. Parameter estimates from these analyses will be compared to estimates from multiple imputation analyses to gauge the impact of including auxiliary variables in the imputation model.
(d) Control-based multiple imputation, in which missing values of the primary outcome for the hearing aid condition without a dementia diagnosis are imputed based on the successful aging control intervention condition. This analysis will investigate the strength of the primary analysis results to the missing at random assumption.

(e) Analysis of cognitive decline based on a categorized version of time (visits) rather than continuous time in years.
(f) A replication of the primary analyses stratified by subgroups of ARIC vs de novo participants. Interaction between intervention condition and recruitment group will be tested in additional models relative to $p < 0.10$.
(g) Exploration of the impact on the primary analysis by further adjustment for additional explanatory variables, as mentioned in section 7.1.

7.5 Secondary Outcomes

An important secondary outcome is time until a composite of (1) adjudicated dementia determined from in-person or phone-based evaluations, (2) adjudicated MCI[26] determined from in-person evaluations (3) a 3-point drop in the 30-item MMSE administered in-person, or (4) a 3-point drop in a factor score derived from the 10-item MMSE orientation subscale and 11-item Blessed scale administered over the phone and rescaled to be equivalent to the 30-item MMSE. Intervention conditions will be compared for the time until the composite outcome utilizing a discrete-time, cause-specific proportional-hazards model with a complimentary log-log link. The same baseline covariates specified for the mixed effects model will be integrated into the proportional-hazards model. Time on study will be the time scale. As a sensitivity analysis, we will examine variations of the composite outcome that include (1) adjudicated MCI from phone-based evaluations and (2) unadjudicated MCI diagnoses identified algorithmically. In exploratory analyses, we will analyze time until (1) adjudicated dementia or MCI diagnosis and (2) decline in MMSE and Blessed scores as separate events.

Three additional secondary outcomes that will be examined include decline in the cognitive domain factor scores for memory, executive function, and language[14] derived from in-person assessments. Intervention conditions will be compared using the same statistical methods described above for the primary outcome. Results from the analysis of the four secondary outcomes will be included in the primary outcome manuscript.

7.6 Exploratory Analyses of the Primary Outcome

a) **Differential practice effects by recruitment source:** The study recruited participants from ARIC and from a community sample. ARIC participants have had prior exposure to testing while non-ARIC participants have not. To address this potential limitation, we will use a mixed effects modeling framework to examine practice effects using an indicator for the first visit in each condition, allowing the practice effect parameter to vary by recruitment source and keeping the estimated intervention effect constant. We will test the fit of that model to one where the intervention effect differs by condition to evaluate whether differential practice effects by recruitment source affects the intervention condition difference. This is a test of effect modification.
b) **Does intervention alter the trajectory of cognitive decline:** In the subset of ARIC participants, we will model prior cognitive change (before the baseline ACHIEVE visit) as well as prospective change through study end (2021-22) using linear spline models, to determine if the rate of change of cognitive decline during ACHIEVE in each condition is different than the rate of change in the preceding years in ARIC visit 5 and 6. We will also test the interaction hypothesis that the hearing intervention deceleration in decline is larger than that in the successful aging intervention condition. The power for analyses in the ARIC subgroup will be lower than optimal since the proportion of ACHIEVE participants recruited from ARIC was smaller than originally anticipated.
c) **Subgroup analyses by demographics:** Given the lower prevalence of hearing loss in women compared to men and blacks compared to other races, an exploratory analysis will be conducted stratifying by sex, race, and education. We will also conduct analyses stratifying by level of hearing loss and level of baseline global cognitive function factor score. Interactions between intervention condition and subgroups will be tested in additional models relative to $p < 0.10$.

d) **Subgroup analyses by AD risk factors:** We will investigate a further refinement of the primary analysis with subgroup analysis for known AD risk factors, including but not limited to ≥ 1 APOE ε4 allele as well as diabetes or hypertension. A subset of ARIC participants will be defined as more likely to be at high risk of AD based on reduced temporal lobe volume meta ROI[27] by structural MRI and no small vessel disease (ARIC Visit 5, 2011-13).

e) **Exclusion of tests with only auditory stimuli:** Among the full study cohort, we will derive a revised global cognitive function factor score excluding tests with only auditory stimuli (logical memory test and digit span backwards). We will use model constraints in a latent variable modeling framework to scale these revised factor scores to be on the same metric as the factor scores in the primary analysis[27].

f) **Mediation by social function:** We will investigate the extent to which cognitive improvements are mediated by improvements *in social function* using causal mediation methods [28].

7.7 Adjustment for Multiple Comparisons

Statistical significance for the primary outcome will be defined as $p < 0.05$. Secondary outcomes will be evaluated for statistical significance with a Hochberg modification to the Bonferroni adjustment, in which the p-values of the five outcomes will be ordered. The largest p-value will be compared relative to $p < 0.05$, and if met, all parameters will be considered significant. If not, then the second largest p-value will be assessed relative to $p < 0.05/2 = 0.025$, and if met then it and all other parameters will be considered significant, and so on for the 3rd p-value compared at 0.05/3=0.017, the fourth compared to 0.05/4 = 0.012, and the fifth compared to 0.05/5 = 0.01.

7.8 Additional Outcomes

Additional outcomes that will be examined include measures of social and physical function, physical activity, and HRQL. Analyses of these outcomes are considered exploratory in nature and will not be viewed as providing confirmatory tests of hypotheses. There will be no adjustment for multiple comparisons and p-values will be provided for descriptive purposes only.

Outcomes will be modeled continuously (outcomes transformed to account for non-normality if necessary) or categorized according to clinically-relevant cut-points[29]. Intervention conditions will be compared using the same approach adopted for the primary analysis.

8. Global cognitive function factor score

The global cognitive function factor score uses all available cognitive test data, has interval-level properties[30], is internally consistent using ARIC data (Cronbach's alpha=0.87), has minimal floor or ceiling effects[31], and demonstrates reliable measurement precision over a broad range of cognitive ability. We have previously demonstrated criterion validity and established cut-points for clinically relevant impairment of the general cognitive performance factor score[31]. We have since extended the harmonization to 26 studies with over 60,000 people, most of which have longitudinal data. Using simulation, we verified the cognitive metric is the same across datasets[31]. We compared precision of our approach with other approaches to combining data using external data: underscoring enhanced precision in a sample of 10,875 persons in 9 datasets, our approach required the smallest sample size to detect cognitive decline with 80% power (N=232) compared to using only the MMSE (N=277) or summarizing available tests into a z-score (N=291)[27, 32]. We further validated the approach against change in hippocampal volume and overall cortical thickness[27]. The approach is consistent with other harmonization techniques and has been used in several other published studies [25, 31-36] including demonstrations that the factors have the same meaning across datasets with different cognitive tests [15, 25, 31-35, 37-40].

9. COVID-19 Considerations

ACHIEVE completed randomization on October 25, 2019 prior to the outbreak of COVID-19. ACHIEVE suspended in-person research visits and began conducting only telephone contacts in March 2020. In July 2020 ACHIEVE initiated a modified version of the in-clinic neurocognitive battery which was collected over the telephone. For ACHIEVE participants who will become due for their final Year 3 study visit beginning in January 2021 while field site clinics are still closed, their final study visit will be a split visit that will comprise both a phone-based component (conducted at the time of their anticipated 36-month visit) AND an in-person component that will be conducted once field site clinics reopen in the future. At these in-person visits, all the data that are expected based on the 36-month visit per the protocol will be collected. There will be a minimum 3-month interval between the occurrence of a 36-month phone visit and when an in-person visit would be scheduled. For this reason some of the participants will have more than 36-months of follow-up time. Participants will be able to receive the other study intervention at the final in-person visit.

During the COVID-19 quarantine, some ACHIEVE participants may have made less use of their hearing aids and social isolation may have impacted their cognitive status. This has the potential to reduce any effect of the intervention.

10. Software and Statistical Programming

Tabulations and statistical analyses will be performed using SAS® Version 9 (or later) or Mplus® version 8.6 (or later) software. All programming will be done by the ACHIEVE Data Coordinating Center (DCC) which is housed in the Collaborative Studies Coordinating Center (CSCC) at the University of North Carolina at Chapel Hill. Standard CSCC statistical computing procedures will be followed. That is, research staff will submit a written statistical computing request to the statistical programming staff. A computing request number will be assigned to the request and information about the request entered into a database. A statistical programmer will then be assigned to undertake the request. Statisticians will review the programs and output. If necessary, changes will be requested in writing. After the programming has been completed to the satisfaction of the staff, all materials will be archived under the request number. Archived materials will include the original computing request, any subsequent changes, the SAS® or Mplus® code as well as any output, including log and list files and datasets created in the request.

11. References

1. Deal JA, Betz J, Yaffe K, et al. Hearing impairment and incident dementia and cognitive decline in older adults: the Health ABC Study. J Gerontol A Biol Sci Med Sci. May 2017;72(5):703-709. doi:10.1093/gerona/glw069

2. Lin FR, Metter EJ, O'Brien RJ, Resnick SM, Zonderman AB, Ferrucci L. Hearing loss and incident dementia. Arch Neurol. Feb 2011;68(2):214-20. doi:68/2/214 [pii] 10.1001/archneurol.2010.362

3. Livingston G, Huntley J, Sommerlad A, Ames D, Ballard C, Banerjee S, Brayne C, Burns A, Cohen-Mansfield J, Cooper C, Costafreda SG, Dias A, Fox N, Gitlin LN, Howard R, Kales HC, Kivimäki M, Larson EB, Ogunniyi A, Orgeta V, Ritchie K, Rockwood K, Sampson EL, Samus Q, Schneider LS, Selbæk G, Teri L, Mukadam N. Dementia prevention, intervention, and care: 2020 report of the Lancet Commission. Lancet. 2020 Aug 8;396(10248):413-446. doi: 10.1016/S0140-6736(20)30367-6. Epub 2020 Jul 30. PMID: 32738937; PMCID: PMC7392084.

4. Loughrey DG, Kelly ME, Kelley GA, Brennan S, Lawlor BA. Association of age-related hearing loss With cognitive function, cognitive impairment, and dementia: a systematic review and meta-analysis. JAMA Otolaryngol Head Neck Surg. 02 2018;144(2):115-126. doi:10.1001/jamaoto.2017.2513

5. Little RJ, Rubin DB. Causal effects in clinical and epidemiological studies via potential outcomes: concepts and analytical approaches. Annu Rev Public Health. 2000;21:121-45. doi: 10.1146/annurev.publhealth.21.1.121. PMID: 10884949.

6. Senn S. Testing for baseline balance in clinical trials. Stat Med. 1994;13(17):1715-1726

7. Colantuoni E, Rosenblum M. Leveraging prognostic baseline variables to gain precision in randomized trials. Stat Med. 2015 Aug 15;34(18):2602-17. doi: 10.1002/sim.6507. Epub 2015 Apr 14. Erratum in: Stat Med. 2017 Nov 30;36(27):4419. PMID: 25872751; PMCID: PMC5018399.

8. Knopman, D., & Ryberg, S. (1989). A verbal memory test with high predictive accuracy for dementia of the Alzheimer type. Archives of Neurology, 46(2), 141-145. doi:10.1001/archneur.1989.00520380041011

9. Wechsler, D. (1987). Wechsler Memory Scale-Revised. San Antonio, Texas: Psychological Corporation.

10. Ryan, J., & Lopez, S. (2001). Wechsler adult intelligence scale-III. In W. Dorfman, & M. Hersen, Understanding psychological assessment. Perspectives on individual differences. New York, NY: Kluwer Academic/Plenum Publishers.

11. Benton, A., & Hamsher, K. (1976). Multilingual Aphasia Examination. Iowa City, IA: University of Iowa.

12. Williams, B., Mack, W., & Henderson, V. (1989). Boston naming test in Alzheimer's disease. Neuropsychologia, 27(8), 1073-1079. doi:10.1016/0028-3932(89)90186-3.

13. Reitan, R. (1958). Validity of the trail making test as an indicator of organic brain damage. Perceptual and Motor Skills, 8, 271-276. doi:10.2466/PMS.8.7.271-276.

14. Gross AL, Power MC, Albert MS, et al. Application of Latent Variable Methods to the Study of Cognitive Decline When Tests Change over Time. *Epidemiol Camb Mass*. 2015;26(6):878-887. doi:10.1097/EDE.0000000000000379.

15. Morris, J. C., Mohs, R. C., Rogers, H., Fillenbaum, G., & Heyman, A. (1988). Consortium to establish a registry for Alzheimer's disease (CERAD) clinical and neuropsychological assessment of Alzheimer's disease. Psychopharmacology bulletin, 24(4), 641–652.

16. Armstrong N, Gitlin L, Parisi J, Carlson M, Rebok G, Gross A. E pluribus unum: Harmonization of physical functioning across intervention studies of middle-aged and older adults. PLoS One. 2017;12(7):e0181746.

17. Chan K, Gross A, Pezzin L, Brandt J, Kasper J. Harmonizing measures of cognitive performance across international surveys of aging using item response theory. Journal of Aging and Health. 2015;27(8):1392-1414.

18. Hayden K, Jones R, Zimmer C, et al. Factor structure of the National Alzheimer's Coordinating Centers uniform dataset neuropsychological battery: An evaluation of invariance between and within groups over time. Alzheimer Disease and Associated Disorders. 2011;25(2):128-137.

19. Park L, Gross A, McLaren D, et al. Confirmatory factor analysis of the ADNI neuropsychological battery. Brain Imaging and Behavior. 2012;6(4):528-539.

20. Klahr S, Levey A, Beck G, et al. The Effects of Dietary Protein Restriction and Blood-Pressure Control on the Progression of Chronic Renal Disease. The New England Journal of Medicine.1994; 330:877-884. DOI: 10.1056/NEJM199403313301301

21. Van Buuren S. Multiple imputation of discrete and continuous data by fully conditional specification. Statistical Methods in Medical Research. 2007; 16(3): p. 219-242.

22. von Hippel P. How many imputations do you need? A two-stage calculation using a quadratic rule. Sociological Methods & Research. 2018: p. 1-20.

23. Crane, P. K., Narasimhalu, K., Gibbons, L. E., Mungas, D. M., Haneuse, S., Larson, E. B., Kuller, L., Hall, K., & van Belle, G. (2008). Item response theory facilitated cocalibrating cognitive tests and reduced bias in estimated rates of decline. Journal of Clinical Epidemiology, 61(10), 1018–27.e9. doi:10.1016/j.jclinepi.2007.11.011

24. Gibbons, L. E., Feldman, B. J., Crane, H. M., Mugavero, M., Willig, J. H., Patrick, D., Schumacher, J., Saag, M., Kitahata, M. M., & Crane, P. K. (2011). Migrating from a legacy fixed-format measure to CAT administration: calibrating the PHQ-9 to the PROMIS depression measures. Quality of Life Research, 20(9), 1349–1357. doi:10.1007/s11136-011-9882-y.

25. Gross AL, Sherva R,Mukherjee S, et al. Calibrating longitudinal cognition in Alzheimer's disease across diverse test batteries and datasets. Neuroepidemiology. 2014;43(3-4):194-205. doi:10.1159/000367970. doi:10.1161/STROKEAHA.114.007847.

26. Knopman DS, Gottesman RF, Sharrett AR, Wruck LM, Windham BG, Coker L, Schneider AL, Hengrui S, Alonso A, Coresh J, Albert MS, Mosley TH Jr. Mild Cognitive Impairment and Dementia Prevalence: The Atherosclerosis Risk in Communities Neurocognitive Study (ARIC-NCS). Alzheimers Dement (Amst). 2016;2:1-11. doi: 10.1016/j.dadm.2015.12.002. PMID: 26949733; PMCID: PMC4772876.

27. Knopman DS, Griswold ME, Lirette ST, et al. Vascular imaging abnormalities and cognition: mediation by cortical volume in nondemented individuals: atherosclerosis risk in communities-neurocognitive study. *Stroke J Cereb Circ*. 2015;46(2):433-440. doi:10.1161/STROKEAHA.114.007847.

28. MacKinnon, D. P. (2008). Multivariate applications series. Introduction to statistical mediation analysis. New Jersey: Taylor & Francis Group/Lawrence Erlbaum Associates.

29. Davis SM. Mixed models for repeated measures with categorical time effects (MMRM). In: Clinical Trials with Missing Data: A Guide for Practitioners. John Wiley & Sons; 2014.

30. Lord FM. The Relation of Test Score to the Trait Underlying the Test. ETS Res Bull Ser. 1952;1952(2):517-549. doi:10.1002/j.2333-8504.1952.tb00926.x.

31. Gross AL, Jones RN, Fong TG, Tommet D, Inouye SK. Calibration and validation of an innovative approach for estimating general cognitive performance. Neuroepidemiology 2014;42(3):144-153. doi:10.1159/000357647.

32. Gross AL, Mungas DM, Crane PK, et al. Effects of education and race on cognitive decline: An integrative study of generalizability versus study-specific results. Psychol Aging. 2015;30(4):863-880. doi:10.1037/pag0000032. ACHIEVE Protocol, v.1.8 November 16, 2017 64.

33. Curran PJ, McGinley JS, Bauer DJ, et al. A Moderated Nonlinear Factor Model for the Development of Commensurate Measures in Integrative Data Analysis. Multivar Behav Res. 2014;49(3):214-231. doi:10.1080/00273171.2014.889594.

34. Schneider BC, Gross AL, Bangen KJ, et al. Association of vascular risk factors with cognition in a multiethnic sample. J Gerontol B Psychol Sci Soc Sci. 2015;70(4):532-544. doi:10.1093/geronb/gbu040.

35. Sisco S, Gross AL, Shih RA, et al. The role of early-life educational quality and literacy in explaining racial disparities in cognition in late life. J Gerontol B Psychol Sci Soc Sci 2015;70(4):557-567. doi:10.1093/geronb/gbt133.

36. Fieo R, Mukherjee S, Dmitrieva NO, et al. Differential item functioning due to cognitive status does not impact depressive symptom measures in four heterogeneous samples of older adults. Int J Geriatr Psychiatry. 2015;30(9):911-918. doi:10.1002/gps.4234.

37. Gross AL, Jones RN, Inouye SK. Development of an Expanded Measure of Physical Functioning for Older Persons in Epidemiologic Research. Res Aging. 2015;37(7):671-694. doi:10.1177/0164027514550834.

38. Purgato M, Gross AL, Jordans MJD, de Jong JTVM, Barbui C, Tol W. Psychosocial interventions for children exposed to traumatic events in low- and middle-income countries: study protocol of an individual patient data meta-analysis. Syst Rev. 2014;3:34. doi:10.1186/2046-4053-3-34.

39. Takane Y, Leeuw J de. On the relationship between item response theory and factor analysis of discretized variables. Psychometrika. 1987;52(3):393-408. doi:10.1007/BF02294363.

40. van der Leeuw G, Eggermont LHP, Shi L, et al. Pain and Cognitive Function Among Older Adults Living in the Community. J Gerontol A Biol Sci Med Sci. 2016;71(3):398-405. doi:10.1093/gerona/glv166.
