# Supplementary material for: Hearing Intervention, Social Isolation, and Loneliness: A Secondary Analysis of the ACHIEVE Randomized Clinical Trial
Source: JAMA Intern Med. 2025 May 12;185(7):797–806. doi: 10.1001/jamainternmed.2025.1140 (PMC12070280; doi:10.1001/jamainternmed.2025.1140)

Supplemental Online Content

Reed NS, Chen J, Huang AR, et al; ACHIEVE Collaborative Research Group. Hearing intervention, social isolation, and loneliness: a secondary analysis of the ACHIEVE randomized clinical trial. *JAMA Intern Med*. Published online May 12, 2025. doi:10.1001/jamainternmed.2025.1140

eTable 1. Demographic and Clinical Characteristics at Baseline of ACHIEVE Participants Stratified by Randomly Assigned Treatment and Recruitment Source (N=977)
eTable 2. Social Network and Loneliness Characteristics of ACHIEVE Participants Stratified by Randomly Assigned Treatment and Recruitment Source (N=977)
eTable 3. Social Network and Loneliness Characteristics of ACHIEVE Participants Stratified by Randomly Assigned Treatment and Recruitment Source (N=977)
eFigure 1. Trial Profile
eFigure 2. Sensitivity Analysis of 3-Year Change in Social Network Characteristics and Loneliness by Randomly Assigned Treatment among the Total Cohort and Stratified by Recruitment Source, Analysis not adjusted for COVID-19 (N=977)
eFigure 3. Sensitivity Analysis of 3-Year Change in Social Network Characteristics and Loneliness by Randomly Assigned Treatment among the Total Cohort and Stratified by Recruitment Source, Per Protocol Analysis (N=824)
eFigure 4. Sensitivity Analysis of 3-Year Change in Social Network Characteristics and Loneliness by Randomly Assigned Treatment among the Total Cohort and Stratified by Recruitment Source, Complier Average Causal Effect Analysis (N=977)
eFigure 5. Sensitivity Analysis of 3-Year Change in Social Network Characteristics and Loneliness by Randomly Assigned Treatment among Female Participants (N=454)
eFigure 6. Sensitivity Analysis of 3-Year Change in Social Network Characteristics and Loneliness by Randomly Assigned Treatment among Male Participants (N=523)

This supplemental material has been provided by the authors to give readers additional information about their work.

© 2025 Reed NS et al. *JAMA Internal Medicine*.

eTable 1. Demographic and Clinical Characteristics at Baseline of ACHIEVE Participants Stratified by Randomly Assigned Treatment and Recruitment Source (N=977)

	ARIC (N=238)			De Novo (N=739)		
	All	Control (N=118)	Intervention (N=120)	All	Control (N=369)	Intervention (N=370)
Age, y, mean (SD) [N]	78.4 (2.9) [238]	78.1 (2.9) [118]	78.8 (2.9) [120]	75.6 (4.0) [739]	76.0 (4.2) [369]	75.2 (3.8) [370]
Female, No. (%)	147/238 (61.8)	73/118 (61.9)	74/120 (61.7)	376/739 (50.9)	186/369 (50.4)	190/370 (51.4)
Race, No. (%)						
White	169/238 (71.0)	83/118 (70.3)	86/120 (71.7)	689/739 (93.2)	341/369 (92.4)	348/370 (94.1)
Black	68/238 (28.6)	35/118 (29.7)	33/120 (27.5)	44/739 (6.0)	24/369 (6.5)	20/370 (5.4)
Other	1/238 (0.4)	0/118 (0.0)	1/120 (0.8)	6/739 (0.8)	4/369 (1.1)	2/370 (0.5)
Center, No. (%)						
Forsyth County, NC	61/238 (25.6)	30/118 (25.4)	31/120 (25.8)	175/739 (23.7)	89/369 (24.1)	86/370 (23.2)
Jackson, MI	63/238 (26.5)	33/118 (28.0)	30/120 (25.0)	180/739 (24.4)	90/369 (24.4)	90/370 (24.3)
Minneapolis, MN	43/238 (18.1)	22/118 (18.6)	21/120 (17.5)	193/739 (26.1)	94/369 (25.5)	99/370 (26.8)
Washington County, MD	71/238 (29.8)	33/118 (28.0)	38/120 (31.7)	191/739 (25.8)	96/369 (26.0)	95/370 (25.7)
Education, No. (%)						
<High school	22/237 (9.3)	10/118 (8.5)	12/119 (10.1)	15/739 (2.0)	8/369 (2.2)	7/370 (1.9)
High school, GED, or vocational school	96/237 (40.5)	48/118 (40.7)	48/119 (40.3)	322/739 (43.6)	164/369 (44.4)	158/370 (42.7)
Some college, graduate, or professional school	119/237 (50.2)	60/118 (50.8)	59/119 (49.6)	402/739 (54.4)	197/369 (53.4)	205/370 (55.4)
Better Ear Pure-Tone Average, db HL, mean (SD) [N]	39.1 (6.7) [238]	38.7 (6.7) [118]	39.5 (6.7) [120]	39.5 (7.0) [739]	39.5 (6.8) [369]	39.6 (7.2) [370]
Quick Speech in Noise Average Score, mean (SD) [N]	18.1 (5.7) [238]	18.0 (5.8) [118]	18.1 (5.6) [120]	18.5 (5.1) [734]	18.5 (4.7) [366]	18.6 (5.4) [368]
Hearing Handicap Inventory for Elderly Score, No. (%)						
None (0-8)	107/237 (45.1)	57/118 (48.3)	50/119 (42.0)	197/733 (26.9)	97/367 (26.4)	100/366 (27.3)
Mild-Moderate (10-24)	103/237 (43.5)	50/118 (42.4)	53/119 (44.5)	384/733 (52.4)	197/367 (53.7)	187/366 (51.1)
Severe (26-40)	27/237 (11.4)	11/118 (9.3)	16/119 (13.4)	152/733 (20.7)	73/367 (19.9)	79/366 (21.6)
Marital Status, No. (%)	137/238 (57.6)	69/118 (58.5)	68/120 (56.7)	465/739 (62.9)	239/369 (64.8)	226/370 (61.1)
Participant Part of a Recruited Spousal Pair, No. (%)	23/238 (9.7)	11/118 (9.3)	12/120 (10.0)	67/739 (9.1)	33/369 (8.9)	34/370 (9.2)
Live Alone, No. (%)	83/231 (35.9)	39/115 (33.9)	44/116 (37.9)	207/737 (28.1)	98/369 (26.6)	109/368 (29.6)
CES-Depression Scale, mean (SD) [N]	2.7 (2.7) [238]	2.7 (2.6) [118]	2.7 (2.9) [120]	2.4 (2.5) [739]	2.4 (2.4) [369]	2.4 (2.6) [370]
Use Anti-depressant, No. (%)	19/238 (8.0)	6/118 (5.1)	13/120 (10.8)	113/739 (15.3)	60/369 (16.3)	53/370 (14.3)
Global Cognition, mean (SD) [N]	-0.379 (1.042) [238]	-0.346 (1.062) [118]	-0.411 (1.025) [120]	0.123 (0.851) [739]	0.096 (0.818) [369]	0.149 (0.883) [370]

Factor scores of global cognition was developed using a validated latent variable modelling approach and standardized to the baseline with higher scores indicating better cognitive function. ARIC=Atherosclerosis Risk in Communities. GED=general educational development credential. dB HL=decibels hearing level.

eTable 2. Social Network and Loneliness Characteristics of ACHIEVE Participants Stratified by Randomly Assigned Treatment (N=977)

	All	Control (N=487)	Intervention (N=490)
Social network size, number of people with regular contact, mean (SD) [N]			
Baseline	22.4 (10.6) [977]	22.3 (10.2) [487]	22.6 (11.1) [490]
Six months	22.6 (10.5) [930]	22.2 (10.3) [459]	23.0 (10.7) [471]
Year One	21.5 (10.4) [933]	21.1 (10.0) [460]	21.9 (10.9) [473]
Year Two	20.3 (10.2) [901]	19.5 (10.1) [445]	20.9 (10.2) [456]
Year Three	20.5 (10.6) [873]	19.8 (10.2) [435]	21.3 (11.0) [438]
Social network diversity, number of high-contact roles, mean (SD) [N]			
Baseline	5.89 (1.64) [977]	5.92 (1.66) [487]	5.87 (1.62) [490]
Six months	5.97 (1.63) [930]	5.88 (1.65) [459]	6.06 (1.61) [471]
Year One	5.77 (1.63) [933]	5.69 (1.65) [460]	5.84 (1.61) [473]
Year Two	5.58 (1.61) [901]	5.46 (1.61) [445]	5.71 (1.61) [456]
Year Three	5.59 (1.67) [873]	5.51 (1.67) [435]	5.68 (1.66) [438]
Embedded social networks, number of network domains, mean (SD) [N]			
Baseline	2.70 (1.65) [977]	2.76 (1.60) [487]	2.63 (1.69) [490]
Six months	2.72 (1.66) [930]	2.70 (1.61) [459]	2.74 (1.70) [471]
Year One	2.55 (1.64) [933]	2.52 (1.58) [460]	2.58 (1.71) [473]
Year Two	2.38 (1.67) [901]	2.28 (1.67) [445]	2.48 (1.66) [456]
Year Three	2.39 (1.69) [873]	2.30 (1.64) [435]	2.48 (1.73) [438]
UCLA Loneliness scale score, mean (SD) [N]			
Baseline	32.7 (8.5) [961]	32.7 (8.6) [482]	32.8 (8.4) [479]
Six months	32.3 (8.7) [918]	33.0 (8.9) [457]	31.7 (8.5) [461]
Year One	32.2 (8.7) [926]	32.6 (8.8) [458]	31.8 (8.6) [468]
Year Two	32.5 (8.7) [899]	33.3 (8.8) [444]	31.8 (8.5) [455]
Year Three	32.9 (8.9) [868]	33.5 (8.8) [434]	32.3 (8.9) [434]

eTable 3. Social Network and Loneliness Characteristics of ACHIEVE Participants Stratified by Randomly Assigned Treatment and Recruitment Source (N=977)

	ARIC (N=238)			De Novo (N=739)		
	All	Control (N=118)	Intervention (N=120)	All	Control (N=369)	Intervention (N=370)
Social network size, number of people with regular contact, mean (SD) [N]						
Baseline	22.0 (10.2) [238]	23.1 (9.7) [118]	21.0 (10.7) [120]	22.6 (10.8) [739]	22.1 (10.3) [369]	23.1 (11.2) [370]
Six months	22.2 (10.3) [230]	23.3 (9.7) [115]	21.1 (10.8) [115]	22.7 (10.6) [700]	21.8 (10.5) [344]	23.6 (10.7) [356]
Year One	21.4 (9.9) [230]	21.9 (9.1) [116]	20.8 (10.7) [114]	21.6 (10.6) [703]	20.8 (10.3) [344]	22.3 (10.9) [359]
Year Two	19.9 (9.6) [218]	20.5 (10.6) [111]	19.2 (8.5) [107]	20.4 (10.3) [683]	19.2 (9.9) [334]	21.5 (10.7) [349]
Year Three	19.9 (9.7) [204]	20.4 (10.0) [105]	19.5 (9.4) [99]	20.7 (10.9) [669]	19.6 (10.3) [330]	21.8 (11.4) [339]
Social network diversity, number of high-contact roles, mean (SD) [N]						
Baseline	5.87 (1.57) [238]	6.13 (1.57) [118]	5.63 (1.55) [120]	5.90 (1.66) [739]	5.86 (1.69) [369]	5.95 (1.64) [370]
Six months	5.90 (1.52) [230]	6.14 (1.46) [115]	5.67 (1.55) [115]	5.99 (1.67) [700]	5.79 (1.71) [344]	6.18 (1.60) [356]
Year One	5.80 (1.54) [230]	5.92 (1.50) [116]	5.68 (1.58) [114]	5.76 (1.66) [703]	5.61 (1.70) [344]	5.89 (1.61) [359]
Year Two	5.50 (1.44) [218]	5.59 (1.57) [111]	5.42 (1.30) [107]	5.61 (1.66) [683]	5.42 (1.62) [334]	5.79 (1.68) [349]
Year Three	5.56 (1.49) [204]	5.70 (1.53) [105]	5.42 (1.44) [99]	5.60 (1.72) [669]	5.45 (1.72) [330]	5.75 (1.71) [339]
Embedded social networks, number of network domains, mean (SD) [N]						
Baseline	2.55 (1.62) [238]	2.86 (1.59) [118]	2.24 (1.59) [120]	2.75 (1.65) [739]	2.73 (1.61) [369]	2.76 (1.70) [370]
Six months	2.56 (1.57) [230]	2.80 (1.52) [115]	2.32 (1.60) [115]	2.77 (1.69) [700]	2.67 (1.65) [344]	2.87 (1.72) [356]
Year One	2.40 (1.54) [230]	2.53 (1.46) [116]	2.25 (1.61) [114]	2.60 (1.68) [703]	2.51 (1.61) [344]	2.68 (1.73) [359]
Year Two	2.17 (1.55) [218]	2.20 (1.72) [111]	2.13 (1.36) [107]	2.45 (1.70) [683]	2.31 (1.65) [334]	2.58 (1.73) [349]
Year Three	2.16 (1.57) [204]	2.26 (1.62) [105]	2.05 (1.51) [99]	2.46 (1.72) [669]	2.31 (1.65) [330]	2.61 (1.77) [339]
UCLA Loneliness scale score, mean (SD) [N]						
Baseline	32.4 (8.5) [234]	31.7 (8.3) [116]	33.1 (8.6) [118]	32.8 (8.5) [727]	33.0 (8.7) [366]	32.7 (8.4) [361]
Six months	32.4 (8.9) [228]	32.4 (8.7) [114]	32.4 (9.0) [114]	32.3 (8.7) [690]	33.2 (9.0) [343]	31.5 (8.3) [347]
Year One	32.2 (8.8) [229]	32.3 (8.7) [116]	32.2 (8.9) [113]	32.2 (8.7) [697]	32.7 (8.9) [342]	31.7 (8.5) [355]
Year Two	32.1 (8.9) [218]	32.5 (8.8) [111]	31.7 (8.9) [107]	32.7 (8.6) [681]	33.5 (8.8) [333]	31.8 (8.3) [348]
Year Three	32.8 (9.0) [203]	32.5 (8.1) [105]	33.2 (9.9) [98]	33.0 (8.8) [665]	33.9 (9.0) [329]	32.1 (8.6) [336]

eFigure 1. Trial Profile

977 Randomized

490 Hearing intervention

N for SNI	Baseline	N for ULS
490	Completed	479
0	Not Completed	11
**N for SNI**	**6 Month Follow-Up**	**N for ULS**
471	Completed	461
0	Not Completed	10
15	Not Collected	15
3	Lost to follow-up	3
1	Withdrew	1
0	Deceased	0
**N for SNI**	**Year-1 Follow-Up**	**N for ULS**
472	Completed	467
0	Not Completed	5
10	Not Collected	10
5	Lost to follow-up	5
1	Withdrew	1
2	Deceased	2
**N for SNI**	**Year-2 Follow-Up**	**N for ULS**
456	Completed	455
0	Not Completed	1
10	Not Collected	10
10	Lost to follow-up	10
4	Withdrew	4
10	Deceased	10
**N for SNI**	**Year-3 Follow-Up**	**N for ULS**
438	Completed	434
0	Not Completed	4
10	Not Collected	10
18	Lost to follow-up	18
6	Withdrew	6
18	Deceased	18

487 Health education control

N for SNI	Baseline	N for ULS
487	Completed	482
0	Not Completed	5
**N for SNI**	**6 Month Follow-Up**	**N for ULS**
459	Completed	457
0	Not Completed	1
10	Not Collected	11
3	Lost to follow-up	3
15	Withdrew	15
0	Deceased	0
**N for SNI**	**Year-1 Follow-Up**	**N for ULS**
460	Completed	458
0	Not Completed	2
5	Not Collected	5
3	Lost to follow-up	3
17	Withdrew	17
2	Deceased	2
**N for SNI**	**Year-2 Follow-Up**	**N for ULS**
445	Completed	444
0	Not Completed	1
10	Not Collected	10
4	Lost to follow-up	4
18	Withdrew	18
10	Deceased	10
**N for SNI**	**Year-3 Follow-Up**	**N for ULS**
434	Completed	433
0	Not Completed	1
9	Not Collected	9
6	Lost to follow-up	6
20	Withdrew	20
18	Deceased	18

Abbreviations: SNI, Cohen Social network index; ULS, UCLA Loneliness score.

eFigure 2. Sensitivity Analysis of 3-Year Change in Social Network Characteristics and Loneliness by Randomly Assigned Treatment among the Total Cohort and Stratified by Recruitment Source, Analysis not adjusted for COVID-19 (N=977)

	3-Year Change β (95% CI)	Difference Between Intervention & Control 3-Year Change β (95% CI)
**Social Network Size (0-84)**		
**Total (N=977)**		
Control	-3.06 (-3.79, -2.32)	1.08 (0.04, 2.12)
Intervention	-1.98 (-2.72, -1.25)	
**ARIC (N=238)**		
Control	-3.69 (-5.40, -1.98)	1.56 (-0.55, 3.68)
Intervention	-2.12 (-3.93, -0.32)	
**De Novo (N=739)**		
Control	-2.98 (-3.85, -2.11)	0.97 (-0.24, 2.18)
Intervention	-2.01 (-2.87, -1.15)	
**Social Network Diversity (0-12)**		
**Total (N=977)**		
Control	-0.52 (-0.64, -0.40)	0.19 (0.02, 0.36)
Intervention	-0.33 (-0.45, -0.21)	
**ARIC (N=238)**		
Control	-0.65 (-0.94, -0.35)	0.30 (-0.07, 0.66)
Intervention	-0.35 (-0.66, -0.03)	
**De Novo (N=739)**		
Control	-0.50 (-0.64, -0.35)	0.14 (-0.06, 0.34)
Intervention	-0.35 (-0.50, -0.21)	
**Social Network Embeddedness (0-8)**		
**Total (N=977)**		
Control	-0.52 (-0.65, -0.40)	0.27 (0.10, 0.45)
Intervention	-0.25 (-0.38, -0.13)	
**ARIC (N=238)**		
Control	-0.74 (-1.02, -0.46)	0.48 (0.14, 0.83)
Intervention	-0.25 (-0.54, 0.04)	
**De Novo (N=739)**		
Control	-0.48 (-0.63, -0.33)	0.21 (0.00, 0.41)
Intervention	-0.27 (-0.42, -0.12)	
**Loneliness (20-80)**		
**Total (N=977)**		
Control	1.06 (0.47, 1.65)	-0.94 (-1.77, -0.11)
Intervention	0.12 (-0.47, 0.71)	
**ARIC (N=238)**		
Control	1.51 (0.10, 2.92)	-0.73 (-2.49, 1.03)
Intervention	0.78 (-0.75, 2.31)	
**De Novo (N=739)**		
Control	1.04 (0.35, 1.73)	-1.05 (-2.00, -0.09)
Intervention	-0.01 (-0.69, 0.67)	

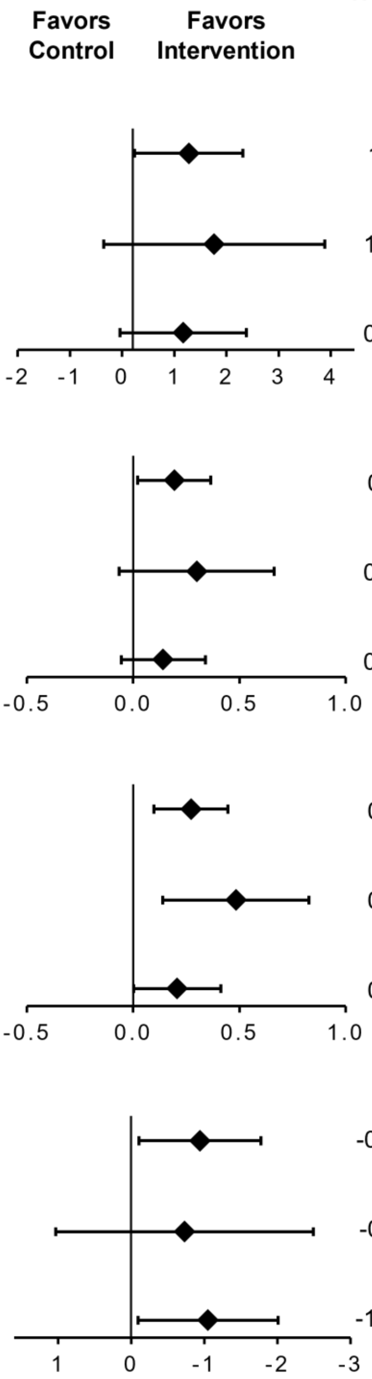

Parameter estimates and 95% Cis were calculated from a linear mixed effects model that adjusted for baseline age, sex, education, field site, better ear pure-tone average, speech-in-noise understanding, hearing handicap inventory for the elderly score, marital status, living alone, global cognition, CES-depression scale, anti-depressant use, and whether the participant was part of a recruited spousal pair. Multiple imputation by chained equations was employed to impute missing covariates.

eFigure 3. Sensitivity Analysis of 3-Year Change in Social Network Characteristics and Loneliness by Randomly Assigned Treatment among the Total Cohort and Stratified by Recruitment Source, Per Protocol Analysis (N=824)

	3-Year Change β (95% CI)	Difference Between Intervention & Control 3-Year Change β (95% CI)
**Social Network Size (0-84)**		
**Total (N=824)**		
Control	-0.69 (-1.79, 0.42)	1.07 (-0.04, 2.18)
Intervention	0.38 (-0.69, 1.46)	
**ARIC (N=204)**		
Control	-2.27 (-4.91, 0.37)	1.82 (-0.43, 4.08)
Intervention	-0.45 (-3.12, 2.23)	
**De Novo (N=620)**		
Control	-0.20 (-1.53, 1.14)	0.84 (-0.45, 2.13)
Intervention	0.65 (-0.65, 1.94)	
**Social Network Diversity (0-12)**		
**Total (N=824)**		
Control	-0.16 (-0.34, 0.03)	0.22 (0.04, 0.41)
Intervention	0.07 (-0.11, 0.25)	
**ARIC (N=204)**		
Control	-0.40 (-0.84, 0.04)	0.36 (-0.02, 0.75)
Intervention	-0.04 (-0.49, 0.41)	
**De Novo (N=620)**		
Control	-0.07 (-0.29, 0.15)	0.16 (-0.06, 0.37)
Intervention	0.09 (-0.13, 0.30)	
**Social Network Embeddedness (0-8)**		
**Total (N=824)**		
Control	-0.12 (-0.31, 0.07)	0.29 (0.10, 0.48)
Intervention	0.17 (-0.02, 0.35)	
**ARIC (N=204)**		
Control	-0.44 (-0.89, 0.00)	0.59 (0.22, 0.96)
Intervention	0.15 (-0.30, 0.60)	
**De Novo (N=620)**		
Control	-0.02 (-0.25, 0.21)	0.19 (-0.03, 0.41)
Intervention	0.17 (-0.06, 0.39)	
**Loneliness (20-80)**		
**Total (N=824)**		
Control	1.45 (0.59, 2.31)	-1.23 (-2.09, -0.37)
Intervention	0.22 (-0.61, 1.05)	
**ARIC (N=204)**		
Control	0.98 (-1.14, 3.12)	-1.35 (-3.16, 0.46)
Intervention	-0.37 (-2.53, 1.79)	
**De Novo (N=620)**		
Control	1.59 (0.57, 2.61)	-1.23 (-2.22, -0.23)
Intervention	0.36 (-0.63, 1.35)	

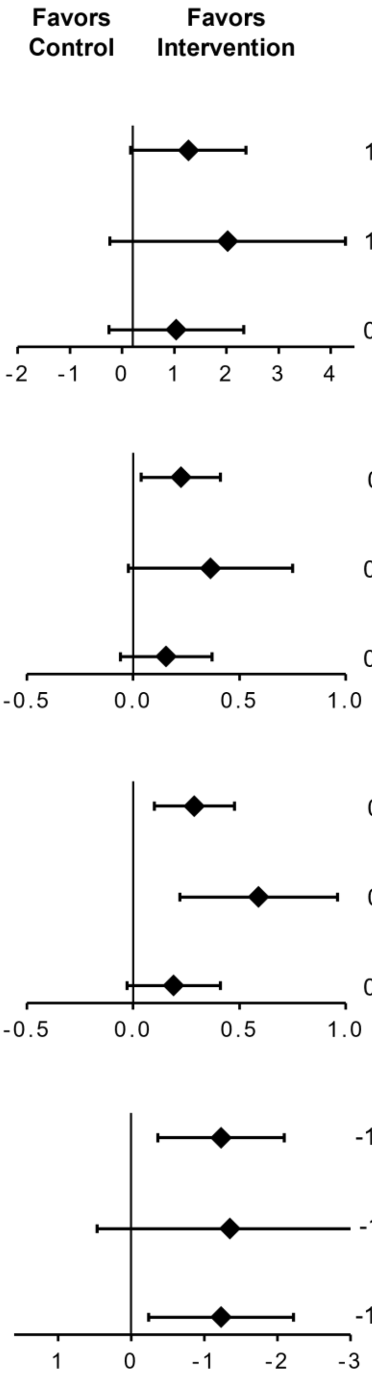

Per protocol analyses were limited to the subset of participants (Total N=824, Control N=391, Intervention N=433) who completed the intervention, had no hearing aid intervention drop-in or drop-out, and had no major protocol deviations. Parameter estimates and 95% Cis were calculated from a linear mixed effects model that adjusted for baseline age, sex, education, field site, better ear pure-tone average, speech-in-noise understanding, hearing handicap inventory for the elderly score, marital status, living alone, global cognition, CES-depression scale, anti-depressant use, whether the participant was part of a recruited spousal pair, and impacts of the COVID-19 pandemics. Multiple imputation by chained equations was employed to impute missing covariates.

eFigure 4. Sensitivity Analysis of 3-Year Change in Social Network Characteristics and Loneliness by Randomly Assigned Treatment among the Total Cohort and Stratified by Recruitment Source, Complier Average Causal Effect Analysis (N=977)

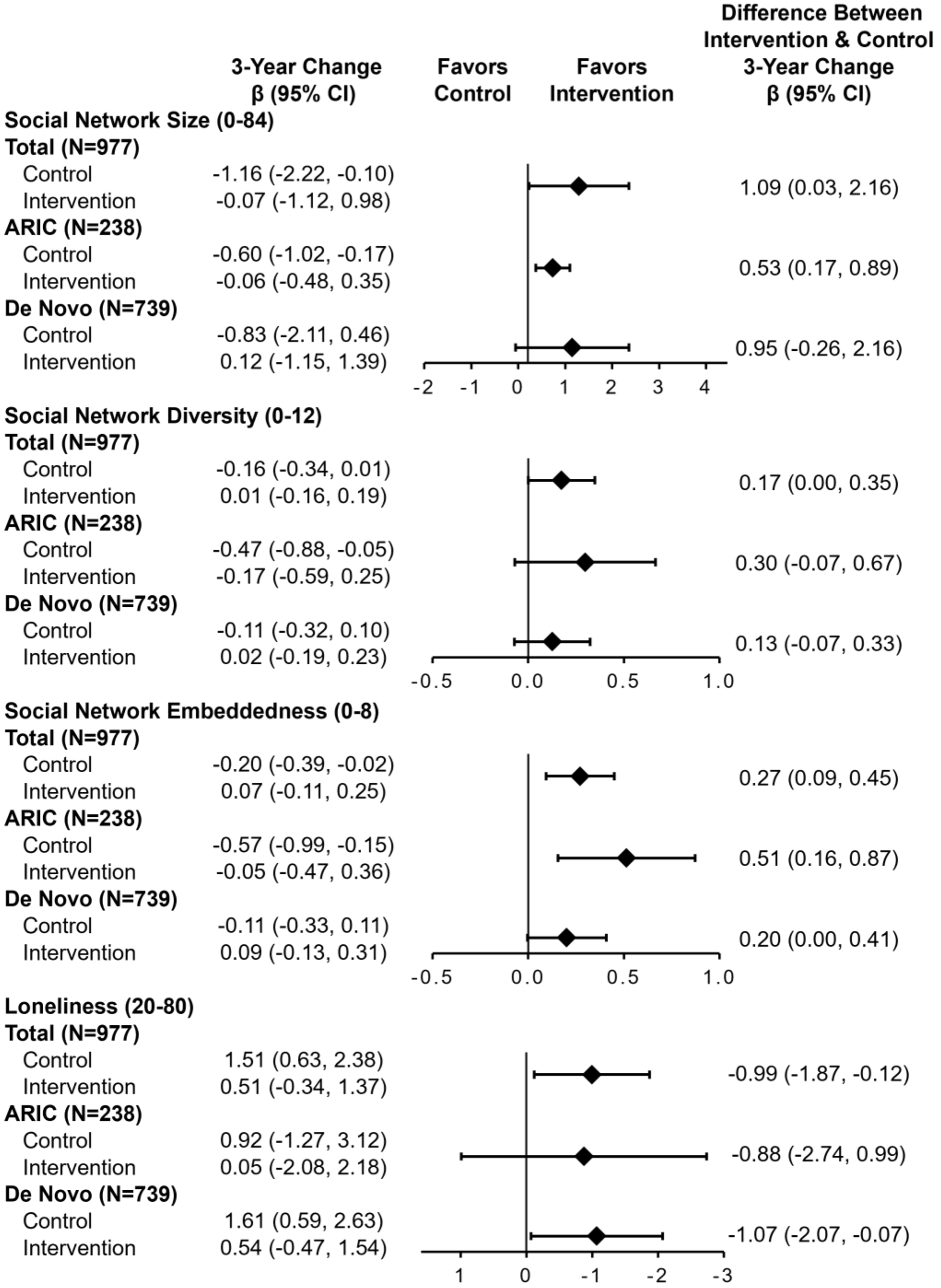

A logistic regression model estimated each participant's probability of being a complier, i.e., adhering to their assigned treatment. An inverse probability weight was subsequently generated and used in the linear mixed effect model. Specifically, the stabilized weight is PN/PD, where PN is predicted via logistic regression using age, sex, field site, and education (c-statistics=0.69), and PD is predicted via logistic regression using baseline age, sex, education, field site, better ear pure-tone average, speech-in-noise understanding, hearing handicap inventory for the elderly score, marital status, living alone, global cognition, CES-depression scale, anti-depressant use, and the four social network and loneliness outcomes. (c-statistics=0.77) Parameter estimates and 95% Cis were calculated from a linear mixed effects model that adjusted for baseline age, sex, education, field site, better ear pure-tone average, speech-in-noise understanding, hearing handicap inventory for the elderly score, marital status, living alone, global cognition, CES-depression scale, anti-depressant use, whether the participant was part of a recruited spousal pair, and impacts of the COVID-19 pandemics. Multiple imputation by chained equations was employed to impute missing covariates.

eFigure 5. Sensitivity Analysis of 3-Year Change in Social Network Characteristics and Loneliness by Randomly Assigned Treatment among Female Participants (N=454)

	3-Year Change β (95% CI)	Difference Between Intervention & Control 3-Year Change β (95% CI)
**Social Network Size (0-84)**		
**Total (N=454)**		
Control	-1.22 (-2.88, 0.43)	1.43 (-0.17, 3.04)
Intervention	0.21 (-1.44, 1.86)	
**ARIC (N=91)**		
Control	-2.35 (-7.19, 2.49)	0.86 (-3.37, 5.09)
Intervention	-1.49 (-6.07, 3.10)	
**De Novo (N=363)**		
Control	-0.93 (-2.83, 0.96)	1.60 (-0.14, 3.35)
Intervention	0.67 (-1.24, 2.58)	
**Social Network Diversity (0-12)**		
**Total (N=454)**		
Control	-0.17 (-0.45, 0.11)	0.26 (-0.01, 0.52)
Intervention	0.08 (-0.19, 0.36)	
**ARIC (N=91)**		
Control	-0.48 (-1.28, 0.32)	0.08 (-0.65, 0.80)
Intervention	-0.40 (-1.14, 0.34)	
**De Novo (N=363)**		
Control	-0.13 (-0.44, 0.19)	0.28 (-0.02, 0.57)
Intervention	0.15 (-0.17, 0.48)	
**Social Network Embeddedness (0-8)**		
**Total (N=454)**		
Control	-0.25 (-0.53, 0.04)	0.33 (0.06, 0.60)
Intervention	0.08 (-0.20, 0.37)	
**ARIC (N=91)**		
Control	-0.33 (-1.14, 0.49)	0.21 (-0.46, 0.89)
Intervention	-0.11 (-0.86, 0.64)	
**De Novo (N=363)**		
Control	-0.21 (-0.53, 0.12)	0.34 (0.03, 0.64)
Intervention	0.13 (-0.20, 0.46)	
**Loneliness (20-80)**		
**Total (N=454)**		
Control	1.43 (0.06, 2.79)	-0.72 (-1.97, 0.54)
Intervention	0.71 (-0.61, 2.03)	
**ARIC (N=91)**		
Control	3.65 (-0.16, 7.46)	-2.35 (-5.44, 0.74)
Intervention	1.30 (-2.33, 4.93)	
**De Novo (N=363)**		
Control	0.87 (-0.68, 2.41)	-0.62 (-2.01, 0.76)
Intervention	0.24 (-1.27, 1.76)	

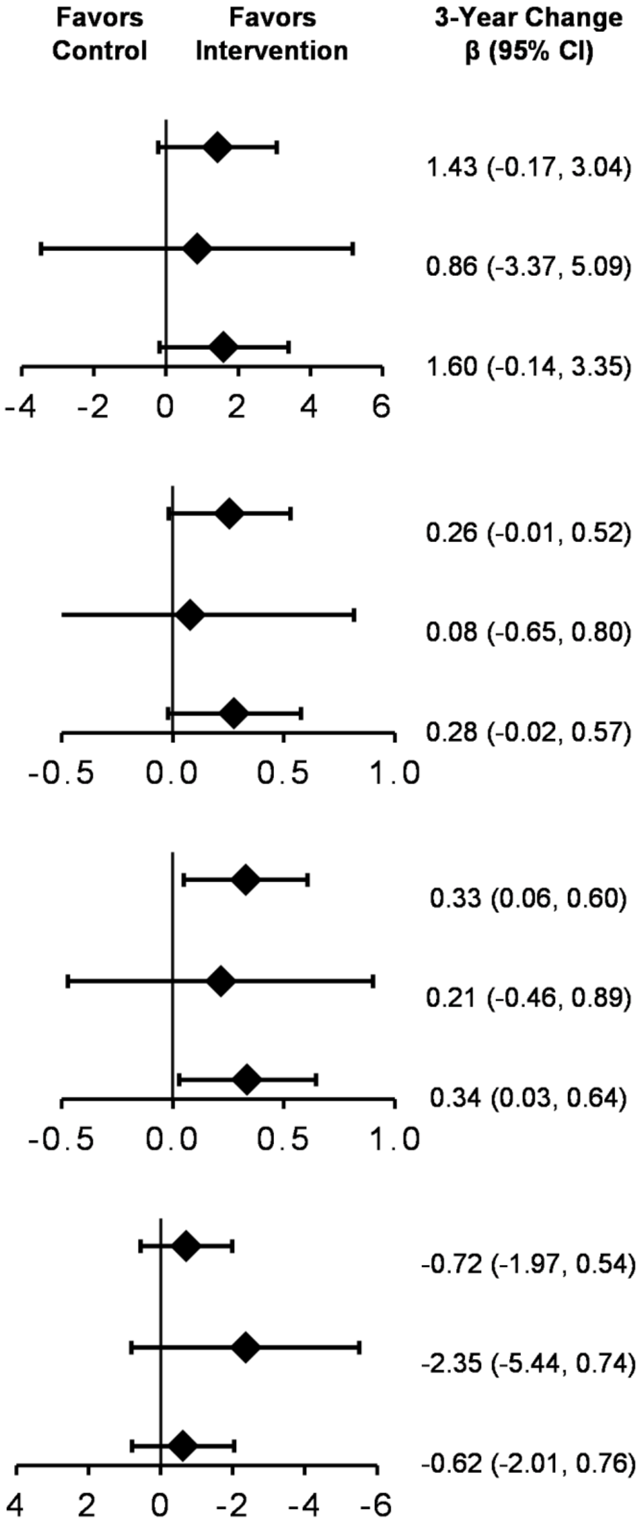

eFigure 6. Sensitivity Analysis of 3-Year Change in Social Network Characteristics and Loneliness by Randomly Assigned Treatment among Male Participants (N=523)

	3-Year Change β (95% CI)	Difference Between Intervention & Control 3-Year Change β (95% CI)
**Social Network Size (0-84)**		
**Total (N=523)**		
Control	-0.37 (-1.78, 1.05)	0.57 (-0.81, 1.95)
Intervention	0.21 (-1.20, 1.61)	
**ARIC (N=147)**		
Control	-2.12 (-4.90, 0.67)	1.77 (-0.71, 4.25)
Intervention	-0.35 (-3.26, 2.57)	
**De Novo (N=376)**		
Control	0.19 (-1.62, 1.99)	0.24 (-1.47, 1.95)
Intervention	0.43 (-1.39, 2.24)	
**Social Network Diversity (0-12)**		
**Total (N=523)**		
Control	-0.13 (-0.36, 0.10)	0.10 (-0.13, 0.33)
Intervention	-0.03 (-0.26, 0.19)	
**ARIC (N=147)**		
Control	-0.35 (-0.83, 0.12)	0.31 (-0.12, 0.74)
Intervention	-0.04 (-0.53, 0.46)	
**De Novo (N=376)**		
Control	-0.03 (-0.32, 0.26)	0.03 (-0.24, 0.31)
Intervention	0.00 (-0.29, 0.29)	
**Social Network Embeddedness (0-8)**		
**Total (N=523)**		
Control	-0.09 (-0.33, 0.15)	0.21 (-0.02, 0.44)
Intervention	0.12 (-0.11, 0.36)	
**ARIC (N=147)**		
Control	-0.61 (-1.07, -0.15)	0.55 (0.15, 0.95)
Intervention	-0.06 (-0.54, 0.42)	
**De Novo (N=376)**		
Control	0.08 (-0.23, 0.39)	0.09 (-0.20, 0.37)
Intervention	0.17 (-0.14, 0.47)	
**Loneliness (20-80)**		
**Total (N=523)**		
Control	1.63 (0.52, 2.74)	-0.96 (-2.09, 0.17)
Intervention	0.67 (-0.44, 1.78)	
**ARIC (N=147)**		
Control	-0.13 (-2.58, 2.32)	-0.42 (-2.66, 1.81)
Intervention	-0.56 (-3.08, 1.97)	
**De Novo (N=376)**		
Control	2.12 (0.77, 3.48)	-1.07 (-2.41, 0.27)
Intervention	1.05 (-0.32, 2.43)	

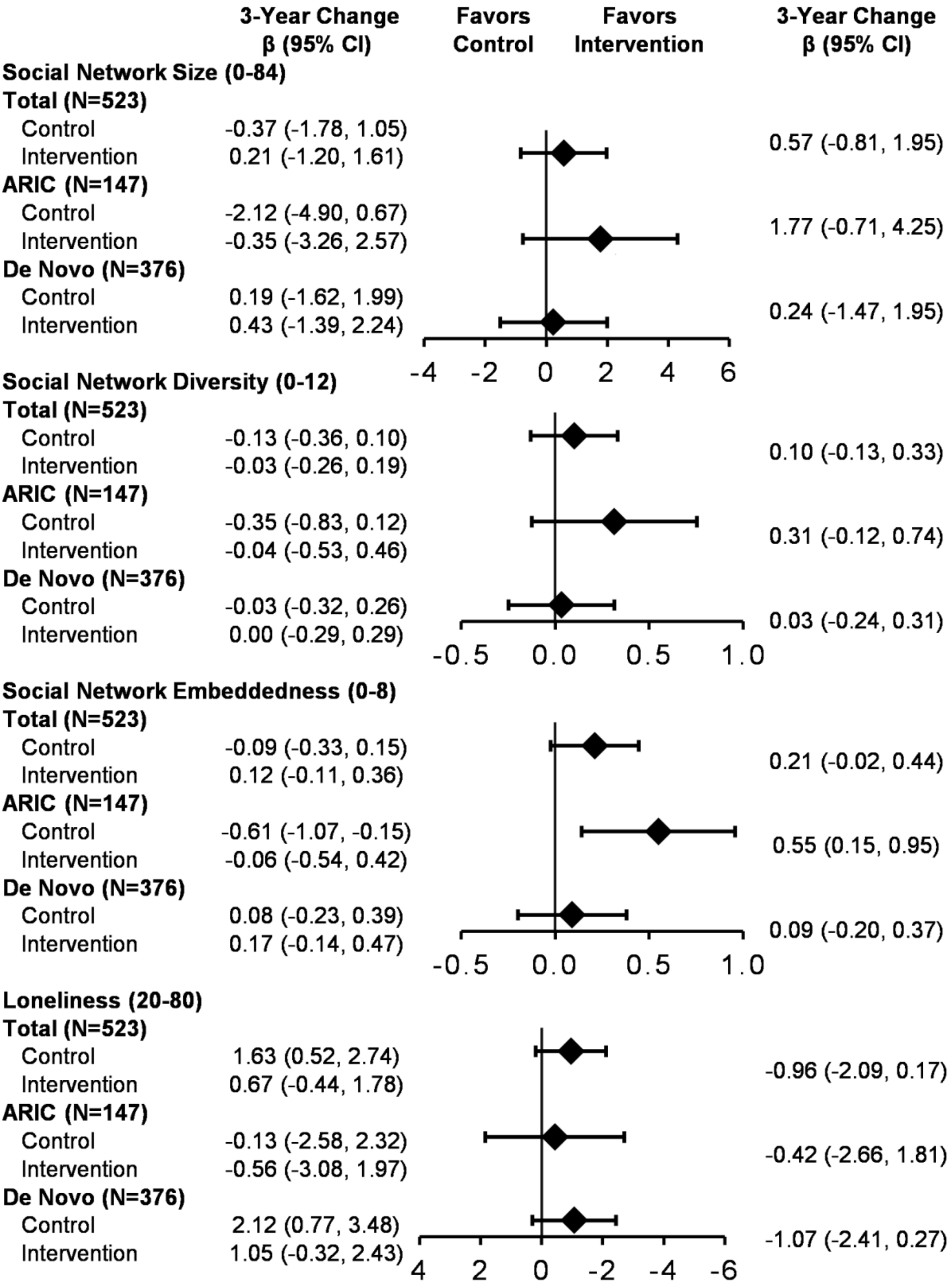

Supplement: Supplement 3. — eTable 1. Demographic and Clinical Characteristics at Baseline of ACHIEVE Participants Stratified by Randomly Assigned Treatment and Recruitment Source (N=977) eTable 2. Social Network and Loneliness Characteristics of ACHIEVE Participants Stratified by Randomly Assigned Treatment and Recruitment Source (N=977) eTable 3. Social Network and Loneliness Characteristics of ACHIEVE Participants Stratified by Randomly Assigned Treatment and Recruitment Source (N=977) eFigure 1. Trial Profile eFigure 2. Sensitivity Analysis of 3-Year Change in Social Network Characteristics and Loneliness by Randomly Assigned Treatment among the Total Cohort and Stratified by Recruitment Source, Analysis not adjusted for COVID-19 (N=977) eFigure 3. Sensitivity Analysis of 3-Year Change in Social Network Characteristics and Loneliness by Randomly Assigned Treatment among the Total Cohort and Stratified by Recruitment Source, Per Protocol Analysis (N=824) eFigure 4. Sensitivity Analysis of 3-Year Change in Social Network Characteristics and Loneliness by Randomly Assigned Treatment among the Total Cohort and Stratified by Recruitment Source, Complier Average Causal Effect Analysis (N=977) eFigure 5. Sensitivity Analysis of 3-Year Change in Social Network Characteristics and Loneliness by Randomly Assigned Treatment among Female Participants (N=454) eFigure 6. Sensitivity Analysis of 3-Year Change in Social Network Characteristics and Loneliness by Randomly Assigned Treatment among Male Participants (N=523) [file jamainternmed-e251140-s003.pdf]
