# Supplementary material for: Hearing Intervention, Social Isolation, and Loneliness: A Secondary Analysis of the ACHIEVE Randomized Clinical Trial
Source: JAMA Intern Med. 2025 May 12;185(7):797–806. doi: 10.1001/jamainternmed.2025.1140 (PMC12070280; doi:10.1001/jamainternmed.2025.1140)
Supplement: Supplement 4. — Nonauthor collaborators [file jamainternmed-e251140-s004.pdf]

Supplemental Online Content: Nonauthor Collaborators
*First name, last name, and suffix (if applicable) are required and will appear in PubMed.

*Group Name(s): ACHIEVE Collaborative Research Group							
***First Name and Middle Initial(s)**	***Last Name**	***Suffix (eg, Jr, III)**	Academic Degrees	Institution	Location (city, state/province, country)	Role or Contribution, eg, chair, principal investigator	Group (if more than 1 Group listed in the byline) and/or Subgroup (eg, Steering Committee)
Marilyn	Albert		PhD	Johns Hopkins University	Baltimore, MD		
Joshua	Betz		MS	Johns Hopkins University	Baltimore, MD		
Alden	Gross		PhD	Johns Hopkins University	Baltimore, MD		
Clarice	Myers		AuD	Johns Hopkins University	Baltimore, MD		
Richey	Sharett		MD, DrPH	Johns Hopkins University	Baltimore, MD		
Melissa	Minotti		MPH	George W. Comstock Center for Public Health Research and Prevention, Johns Hopkins University	Hagerstown, MD		
Spencer	Bolton			George W. Comstock Center for Public Health Research and Prevention, Johns Hopkins University	Hagerstown, MD		
Laura	Sherry		AuD	George W. Comstock Center for Public Health Research and Prevention, Johns Hopkins University	Hagerstown, MD		
Sarah	Aguilar		MS	Univesity of Minnesota	Minneapolis, MN		
Elizabeth	Anderson		PhD, AuD	Univesity of Minnesota	Minneapolis, MN		
Sydney	Boelter			Univesity of Minnesota	Minneapolis, MN		
Elizabeth	Penland Miller			Univesity of Minnesota	Minneapolis, MN		
Debbie	Ng		MPH	Univesity of Minnesota	Minneapolis, MN		
Kristi	Oeding		PhD, AuD	Univesity of Minnesota	Minneapolis, MN		
Sandra	Potter			Univesity of Minnesota	Minneapolis, MN		
Katherine	Teece		AuD	Univesity of Minnesota	Minneapolis, MN		
Soni	Uccellini			Univesity of Minnesota	Minneapolis, MN		
Matthew	Waggenspack		AuD	Univesity of Minnesota	Minneapolis, MN		
Luanne	Welch		RN	Univesity of Minnesota	Minneapolis, MN		
Jacqueline	Weycker		AuD	Univesity of Minnesota	Minneapolis, MN		
Kerry	Witherell		AuD	Univesity of Minnesota	Minneapolis, MN		

Supplemental Online Content: Nonauthor Collaborators
*First name, last name, and suffix (if applicable) are required and will appear in PubMed.

***First Name and Middle Initial(s)**	***Last Name**	***Suffix (eg, Jr, III)**	Academic Degrees	Institution	Location (city, state/province, country)	Role or Contribution, eg, chair, principal investigator	Group (if more than 1 Group listed in the byline) and/or Subgroup (eg, Steering Committee)
Kevin	Sullivan		PhD	University of Mississippi Medical Center	Jackson, MS	Site PI	
Latilraka	Anderson		MS EdS	University of Mississippi Medical Center	Jackson, MS		
Jillian	Burt		MBA	University of Mississippi Medical Center	Jackson, MS		
April	Carr			University of Mississippi Medical Center	Jackson, MS		
Arkenya	Carter		MS	University of Mississippi Medical Center	Jackson, MS		
Sarah	Faucette		PhD, AuD	University of Mississippi Medical Center	Jackson, MS		
Rachel	Foster		MS	University of Mississippi Medical Center	Jackson, MS		
Ceola	Greenwood		MS	University of Mississippi Medical Center	Jackson, MS		
Temeka	Griffin		DPC	University of Mississippi Medical Center	Jackson, MS		
Candace	Jones		MSW EdS	University of Mississippi Medical Center	Jackson, MS		
Dawn	McLendon		LPN	University of Mississippi Medical Center	Jackson, MS		
Stacee	Naylor		MSN RN CCRP	University of Mississippi Medical Center	Jackson, MS		
Jenny	Newman		MS	University of Mississippi Medical Center	Jackson, MS		
Deidre	O'Connor		MS	University of Mississippi Medical Center	Jackson, MS		
Tiffany	Owens		MSCP	University of Mississippi Medical Center	Jackson, MS		
Jeraline	Sims		RN MSN	University of Mississippi Medical Center	Jackson, MS		

Supplemental Online Content: Nonauthor Collaborators
*First name, last name, and suffix (if applicable) are required and will appear in PubMed.

***First Name and Middle Initial(s)**	***Last Name**	***Suffix (eg, Jr, III)**	Academic Degrees	Institution	Location (city, state/province, country)	Role or Contribution, eg, chair, principal investigator	Group (if more than 1 Group listed in the byline) and/or Subgroup (eg, Steering Committee)
Allison	Thweatt		RN	University of Mississippi Medical Center	Jackson, MS		
Tamikia	Washington			University of Mississippi Medical Center	Jackson, MS		
Bria	Backman			Wake Forest University	Winston-Salem, NC		
Debbie	Barr			Wake Forest University	Winston-Salem, NC		
Joshua	Evans			Wake Forest University	Winston-Salem, NC		
Jamie	Hampton		AuD	Wake Forest University	Winston-Salem, NC		
Hailley	Humphrey-Rutledge		AuD	Wake Forest University	Winston-Salem, NC		
Kaila	Liou		AuD	Wake Forest University	Winston-Salem, NC		
Ashley	Mitchell			Wake Forest University	Winston-Salem, NC		
Susan	Smith			Wake Forest University	Winston-Salem, NC		
Nadine	Shelton			Wake Forest University	Winston-Salem, NC		
Ann	Eddins		PhD MBA	University of South Flordia	Tampa, FL		
Emily	Moore		AuD	University of South Flordia	Tampa, FL		
Haley	Neil		AuD	University of South Flordia	Tampa, FL		
Preyanca	Oree		AuD	University of South Flordia	Tampa, FL		
Laura	Westermann		MA	University of South Flordia	Tampa, FL		
Yurun	Cai		PhD	University of Pittsburgh	Pittsburgh, PA		
David	Li		MS	University of North Carolina	Chapel Hill, NC		
Clifford R	Jack		MD	Mayo Clinic	Rochester, MN	Site PI	
David	Knopman		MD	Mayo Clinic	Rochester, MN	Co-Investigator	
Denise	Reyes			Mayo Clinic	Rochester, MN		
AJ	Spychalla			Mayo Clinic	Rochester, MN		
Kaely	Thostenson			Mayo Clinic	Rochester, MN		
Doug	Galasko		PhD	University of California San Diego	San Diego, CA	Chair	Data and Safety Monitoring Board
Julie	Buring		ScD	Harvard University	Cambridge, MA		Data and Safety Monitoring Board
Judy	Dubno		PhD	Medical University South Carolina	Chareston, SC		Data and Safety Monitoring Board

Supplemental Online Content: Nonauthor Collaborators
*First name, last name, and suffix (if applicable) are required and will appear in PubMed.

***First Name and Middle Initial(s)**	***Last Name**	***Suffix (eg, Jr, III)**	Academic Degrees	Institution	Location (city, state/province, country)	Role or Contribution, eg, chair, principal investigator	Group (if more than 1 Group listed in the byline) and/or Subgroup (eg, Steering Committee)
Tom	Greene		PhD	University of Utah	Salt Lake City, UT		Data and Safety Monitoring Board
Lawrence	Lustig		MD	Columbia University	New York, NY		Data and Safety Monitoring Board
Coryse	St. Hillaire-Clarke		PhD	National Institute on Aging	Bethesda, MD		Funding Agency
Matt	Sutterer		PhD	National Institute on Aging	Bethesda, MD		Funding Agency
