# Supplementary material for: Hearing Intervention, Social Isolation, and Loneliness: A Secondary Analysis of the ACHIEVE Randomized Clinical Trial
Source: JAMA Intern Med. 2025 May 12;185(7):797–806. doi: 10.1001/jamainternmed.2025.1140 (PMC12070280; doi:10.1001/jamainternmed.2025.1140)
Supplement: Supplement 5. — Data sharing statement [file jamainternmed-e251140-s005.pdf]

Data Sharing Statement

Reed. Hearing Intervention, Social Isolation, and Loneliness. *JAMA Intern Med*. Published May 12, 2025. doi:10.1001/jamainternmed.2025.1140

Data
Additional Information: ACHIEVE, Clinicaltrials.gov Identifier: NCT03243422
Data available: Yes
Data types: Deidentified participant data
How to access data: BIOLNCC
When available: beginning date: 12-31-2024

Supporting Documents
Document types: None

Additional Information
Who can access the data: researchers whose proposed use of the data has been approved
Types of analyses: for any purpose
Mechanisms of data availability: after approval of a proposal
